# Proteome-wide mapping of PQS-interacting proteins in *Pseudomonas aeruginosa*†
†Electronic supplementary information (ESI) available: Synthetic procedures and data, biological evaluations, chemical proteomics procedures and data. See DOI: 10.1039/c7sc04287f


**DOI:** 10.1039/c7sc04287f

**Published:** 2018-01-19

**Authors:** Rambabu Dandela, Danielle Mantin, Benjamin F. Cravatt, Josep Rayo, Michael M. Meijler

**Affiliations:** a Dept. of Chemistry , The National Institute for Biotechnology in the Negev , Ben-Gurion University of the Negev , Be'er Sheva , Israel . Email: peprayo@gmail.com ; Email: meijler@bgu.ac.il; b The Skaggs Institute for Chemical Biology , Department of Molecular Medicine , The Scripps Research Institute , 10550 North Torrey Pines Road , La Jolla , CA 92037 , USA

## Abstract

The opportunistic human pathogen *Pseudomonas aeruginosa* secretes 2-heptyl-3-hydroxy-4-quinolone (PQS), a quorum sensing (QS) signal that regulates the expression of numerous virulence genes. Here we report the development and application of chemical probes to globally map quinolone binding proteins. The revealed quinolone interactome contains both known as well as newly identified virulence factors and presents new targets for the treatment of bacterial infections.

## Abstract 2

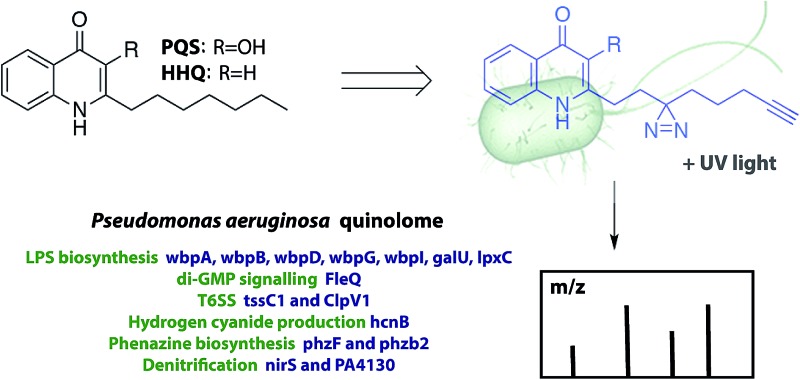
Development and application of chemical probes to globally map key virulence proteins of pathogenic bacteria.

## Introduction

The emergence of Multi-Drug-Resistant (MDR) bacteria increasingly forms a major and global public health issue. Antibiotic resistance alone is predicted to kill 10 million people by 2050.^1^ Even though there is an urgent need for new avenues to treat bacterial pathogens, currently there is a decrease in the rate of introduction of new antibiotics, while their use is constantly increasing.^2^ Such a new avenue, which has gained increasing interest in recent years, is the development of tools to target bacterial virulence mechanisms instead of growth. Targeting such molecular targets would provide less selective pressure among bacteria to gain resistance.^3^–^6^ Bacterial pathogens regulate their virulence primarily in accordance with their population density *via* complex Quorum-Sensing (QS) regulatory networks. This involves the synthesis, secretion, and sensing of small signalling molecules – termed autoinducers – which activate the expression of a myriad of genes that are essential for an efficient colonization of their host environment.^7^ In recent years, an improved understanding of the connection between QS, pathogenicity and the host environment has aided the development of increasingly effective methods to attenuate bacterial virulence. These strategies involve disabling QS with small molecules which target either the sensing,^8^ biosynthesis,^9^ and/or degradation of several autoinducers.^10^ Several well-characterised protein targets have been identified for different autoinducers in different bacteria that provide an initial basis for explaining their cellular activities; however, in spite of these advances, the molecular targets and underlying modes of action remain largely uncharacterised.

**Scheme 1:**
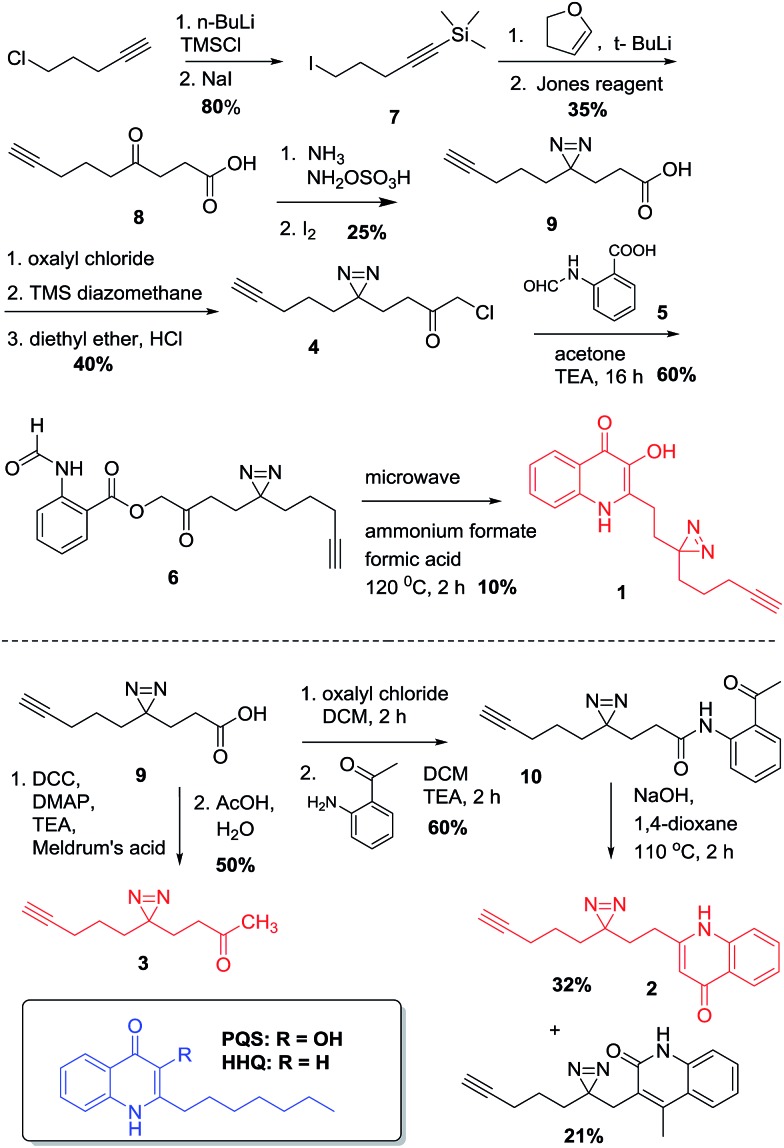
Synthesis of PQS- and HHQ-based probes **1** and **2** and control probe **3**.

*Pseudomonas aeruginosa* is an opportunistic human pathogen that is known to be responsible for a large variety of serious and often life-threatening diseases. Its pathogenicity is strongly related to the production of an unusually large number of virulence factors, which cause tissue damage, delayed airway epithelium wound repair, highly efficient biofilm formation and suppressed innate immune responses.^11^ In this bacterium, one class of key compounds involved in virulence regulation are quinolones, such as 2-heptyl-3-hydroxy-4(1*H*)-quinolone (pseudomonas quinolone signal, PQS) and its direct precursor 2-heptyl-4(1*H*)-quinolone (HHQ) (Scheme 1).^12^ This network, the PQS QS system, has been extensively studied, both *in vitro* and in animal models of infection, yet the precise role played by each component, HHQ and PQS, is not well understood, and recent studies have pointed to a remarkable flexibility of this system, depending on changing conditions. In order to understand the success of *P. aeruginosa* in adapting to changing conditions and environments, we surmise that it is essential to develop a much deeper understanding of the quinolone QS networks. To this end, we, and others,^5^,^13^,^14^ have developed probes where bacterial autoinducers are incorporated with features that enable target identification and quantification. These probes contain the photoreactive diazirine group, to allow for covalent cross-linking of the probe to target proteins, and an alkyne moiety for conjugation to an azide reporter tag through click chemistry, which enables facile detection and/or enrichment of autoinducer-interacting proteins. Here, we address these challenges by first developing an efficient synthetic route to generate several, biologically active, quinolone analogues and then applying these probes to globally assess PQS/HHQ targets in *P. aeruginosa*. These studies are the first to demonstrate that PQS and HHQ not only regulate the transcription, but also *directly interact* with several key virulence pathways. As well as improving our current understanding of bacterial communication and group behaviour, this study has also the potential to uncover new drug targets for combating antibacterial resistance.

## Results and discussion

### Design and synthesis

The design of PQS and HHQ photo-affinity probes follows the principle that introduction of any bulky moiety to the ligand will strongly affect its interaction with potential cognate targets. With this consideration in mind, we designed and synthesized two different probe scaffolds that contain a diazirine photo-cross-linker and a small alkyne handle (Scheme 1). The choice of diazirine as a reactive group was mostly due to its small size, yielding both minimal structural changes to the PQS or HHQ mimic (allowing maximal receptor recognition) and enabling relatively mild irradiation conditions. Both modifications were incorporated into the alkyl side chain of the probes, both due to synthetic considerations and the likelihood that structural modifications to this moiety are better tolerated by PQS/HHQ binders than modifications to the quinolone epitope. The synthesis of the probes with the same alkyl side chain length as their cognate compounds proved to be challenging due to the low stability of one of the intermediates (*i.e.* 4-iodo-1-(trimethylsilyl)-1-butyne). We therefore decided to synthesize analogues with a longer side chain (8 carbons) since this modification has been shown to retain its biological activity, and more importantly, it allowed us to use a more stable intermediate (5-iodo-1-(trimethylsilyl)-1-pentyne). Both diazirine-alkyne PQS and HHQ probes were synthesized by using modified methods of diazirine and quinolone synthesis, with some clear distinctions (see ESI† for details). Briefly, diazirine chloromethyl ketone **4** was used to alkylate *N*-formyl anthranilic acid **5** to provide β-ketodiester **6**. The β-ketodiester **6** was cyclized in excess ammonium formate (NH_4_HCO_2_) and formic acid (HCO_2_H), using microwave irradiation to provide PQS probe **1** (Scheme 1). HHQ probe **2** was synthesized largely following the methodology developed by Pesci *et al.*^15^

### Validation

To test if and how the structural modifications influence the activity of the PQS and HHQ probes, we used a well-established β-galactosidase reporter gene assay in *E. coli* containing the plasmid pEAL08-2, which encodes PqsR under the control of the tac promoter and the β-galactosidase reporter gene lacZ controlled by the pqsA promoter.^16^ We also tested the activities of both probes in a *P. aeruginosa* PQS-reporter strain that is not able to synthesize PQS or HHQ (Δ*pqsAH*). Encouragingly, the activities of both probes proved to be almost identical to their cognate ligands (Fig. S1–S3†). In agreement with previously reported data, PQS, and its corresponding probe, stimulated the PqsR-dependent pqsA transcription in the nanomolar range. On the other hand, HHQ and **2** displayed a more modest stimulation. In order to validate the chemoproteomic approach, we first performed preliminary labelling experiments using the previous reporter strain. This system overexpresses PqsR, the primary PQS binder, and therefore, a relatively clean labelling is expected. Cells were treated with each of the probes, briefly irradiated, lysed, and coupled to a rhodamine-azide tag, using click chemistry.

**Fig. 1:**
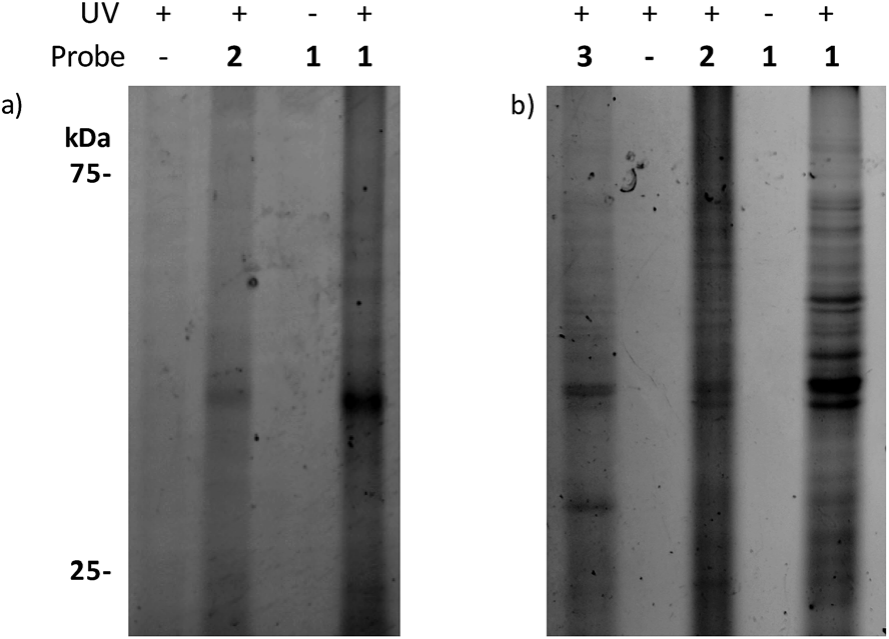
Gel-based profiling of quinolone-binding proteins. SDS-PAGE analysis of: (a) DH5α-pEAL08-2; (b) *P. aeruginosa* PAO1.

When the samples were analysed by SDS-PAGE, robust labelling of a protein with an apparent molecular weight of roughly 40 kDa (calculated molecular weight for ligated full-length PqsR is 37.2 kDa) was observed in the presence of **1** (Fig. 1a). In contrast, this band was clearly reduced in the presence of **2** in accordance with the lower affinity of HHQ for PqsR, and absent or reduced in negative control experiments such as labeling without probe, without UV irradiation, or in a *P. aeruginosa* Δ*pqsAHR* mutant (Fig. S3†). As mentioned, we ultimately aimed to unravel the quinolone interacting proteome in *P. aeruginosa*. Towards this end, we applied the same conditions used in the *E. coli* reporter strain assays in wild-type *P. aeruginosa* cultures. Encouragingly, as seen in Fig. 1b, incubation of intact cells with **1** showed strong labelling of several proteins. Also, similar to the previous experiments, labelling was abolished upon omission of the probe or in absence of UV irradiation. In addition, labelling was reduced in the presence of the HHQ probe, **2**, and markedly reduced for the non-quinolone lipid probe containing alkyne and diazirine moieties embedded in decanone structure **3**. This probe was synthesized in order to help distinguish specific quinolone-interactors from non-specific binders that have strong affinity for the hydrophobic moieties of our quinolone probes.

### Chemical proteomics

**Fig. 2:**
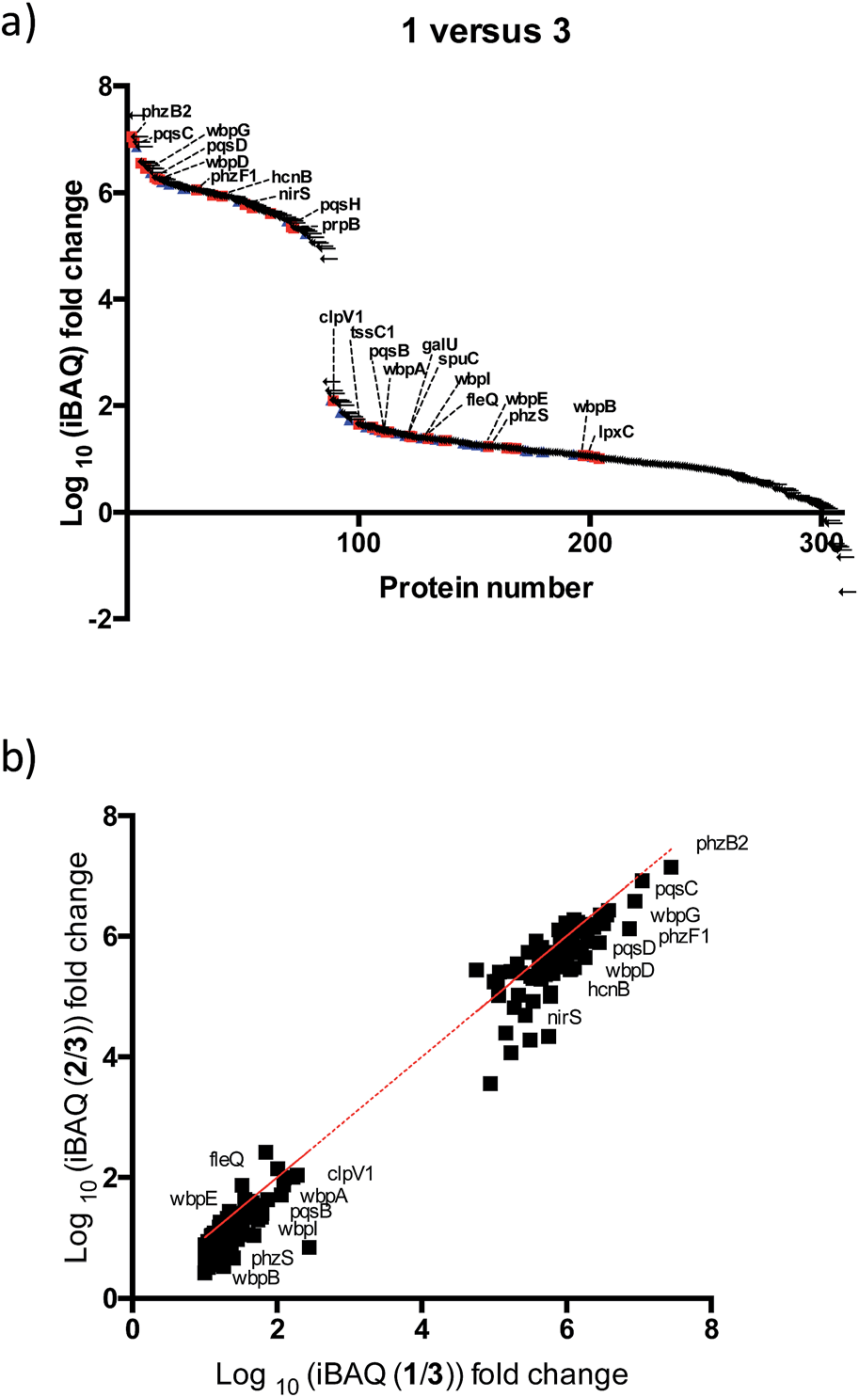
Mass spectrometry based profiling of quinolone-interacting proteins in *P. aeruginosa*. (a) Enrichment plots for total proteins identified in which we compared labelling profiles of **1***versus***3** (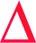 known virulent proteins, 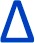 redox enzymes). (b) Comparison of the iBAQ intensity of **1***versus***2**.

**Fig. 3:**
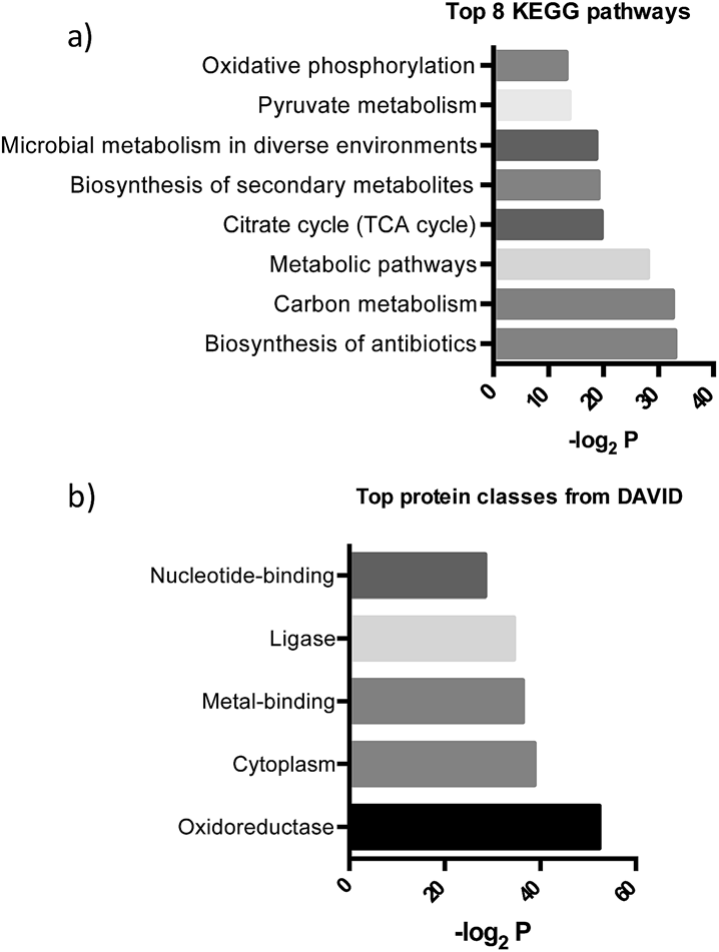
(a) Pathways determined by searching the pulled down proteins in the KEGG database, and (b) biological keywords determined using the DAVID gene ontology server.

After further optimizing labeling conditions, we performed liquid chromatography-tandem mass spectrometry (LC-MS/MS) analyses in wild-type *P. aeruginosa* cultures in order to identify proteins that specifically interact with our probes. First, photolabeled proteins were biotinylated using CuAAC reactions between the alkyne moiety on the probe and biotin azide, followed by enrichment of samples *via* streptavidin-based affinity chromatography. Then, the pulled down proteins were on-bead digested with trypsin and the resulting tryptic peptide mixture was analyzed by liquid chromatography-mass spectrometry. This strategy has been used successfully in recent years to isolate and identify very low abundant proteins from complex proteomes.^17^–^19^ Proteins that exhibited a strong enrichment, log_10_(intensity-based absolute quantification, iBAQ) > 1, for **1***versus* the DMSO negative control were designated as true probe interactors. iBAQ intensities have been proven to provide an accurate proxy for protein abundance.^25^ About 308 proteins met these criteria (ESI Table 1†). Nearly 207 of the identified probe-binding proteins showed strong enrichment when using the quinolone probe, as compared to a non-quinolone lipid probe (Fig. 2a). It should be noted that, as with any chemical proteomics strategy, some of these hits are false positives (*i.e.* proteins that have affinity for the probe, linker, resin or are secondary protein binders but are not ‘true’ PQS or HHQ binders). Interestingly, we also did not observe PqsR enrichment, but could be expected given the fact that at high PQS concentrations the expression of this receptor is strongly down-regulated.^26^ Still, encouragingly, using this methodology we were able to detect all the proteins involved in the biosynthesis of these quinolone virulence factors, with the exception of PqsA. Taking into account that these enzymes are likely to interact with quinolones since they are involved in their biosynthesis, these hits strongly corroborate the methodology presented here. In addition, another key virulence regulator, phzB (involved in pyocyanin biosynthesis), was also identified. A more detailed functional annotation, using the automated Kyoto Encyclopedia of Genes and Genomes (KEGG) pathways^20^ and Database for Annotation, Visualization and Integrated Discovery (DAVID)^21^ analysis tools, helped us to identify several types of proteins unrelated to the canonical PQS pathways, including biosynthetic enzymes responsible for the production of antibiotics and secondary metabolites (Fig. 3a). In-depth analysis of these datasets revealed, perhaps not surprisingly given the metal chelating activity of quinolones,^22^ that both several important oxidoreductases as well as metal binding proteins appear to be able to recognize the PQS/HHQ motif (Fig. 3b).

### Virulence factors are a major part of the quinolone interactome

PQS is known to regulate the expression of a set of 182 genes from which a significant number are PqsR-independent.^23^ From this set of proteins we detected 11% in our studies, suggesting that many of these proteins are regulated by PQS at both the transcriptional and post-transcriptional (direct binding) levels. A broader survey of the identified proteins suggests that PQS interacts with previously unknown virulence factors either directly or through affecting their biosynthetic machinery. For instance, several proteins involved in lipopolysaccharide (LPS) biosynthesis were detected: (a) B-band of O-antigen (wbpA, wbpB, wbpD, wbpG, wbpI); (b) LPS core (galU); (c) lipid A (lpxC). We were also able to identify FleQ, a main effector for di-GMP signalling, which controls the important transition of this bacterium from planktonic to sessile biofilm forms. Another notable finding is that two proteins (tssC1 and ClpV1) involved in the type 6 secretion (T6SS) machinery appear to interact with PQS/HHQ. TTsC1 is involved in biofilm-specific antibiotic resistance,^24^ was shown to be down-regulated in response to pqsE induction.^23^ In addition, several virulence, and resistance pathways previously known to be transcriptionally regulated by PQS were also detected: (a) hydrogen cyanide production (hcnB), (b) phenazine biosynthesis (phzF and phzb2), (c) denitrification (nirS, PA4130), (d) putrescine degradation (spuC), (e) aminoglycoside metabolism (prpB).

Our two probes (1 and 2) also enabled us to compare the relative proteome binding profiles PQS and HHQ. Unexpectedly, in disagreement with a previous transcriptomic study where only 3 genes were shown to be controlled to a significant degree by HHQ,^23^ we did not observe strong differences between the two probes, although the measured iBAQ values,^25^ and thus the captured protein concentrations, appear to be slightly higher for the PQS probe (Fig. 3b). Therefore, these data suggest that even though HHQ only transcriptionally controls the PQS signalling pathways, from a post-transcriptional “point of view” the activity of this autoinducer appears to be far more promiscuous. Understanding these differences will be part of future studies.

In the course of preparation of this manuscript a valuable similar study was published by Spring and coworkers,^27^ in which different PQS- and HHQ-based probes (with azide-containing quinolones) were evaluated in *P. aeruginosa*. Interestingly, we identified all the proteins from the former article, with the exception of the RhrR transcriptional regulator and the protease Pfpl, suggesting that there is a significant overlap in the proteome coverage between the different probes. However, there are some key differences, such as the number of proteins identified in our study, 182, against 11 in the former one. These differences can most likely be attributed to the alternative probe designs. Indeed, differences in labelling using diazirine and azide photocrosslinkers have previously been reported.^28^,^29^ Future binding studies with identified receptors should clarify these differences further.

## Conclusions

We have identified both known and previously unknown protein targets of PQS and HHQ in living bacterial cells using a chemical proteomic strategy that utilizes affinity-based photo-cross-linking probes. Our results demonstrate that our probes mimic the activity of their parent compounds using a well-established quinolone reporter strain. One of the most interesting finding to emerge from our studies is the remarkable promiscuity displayed by both PQS and HHQ to affect myriad pathways, some of which are known to play a key role in the pathogenicity and adaptability of this ubiquitous organism. Projecting forward, it will be highly valuable to experimentally validate the major quinolone interacting proteins unveiled here and to apply this methodology to unravel the mechanism of action of these molecules in other biological systems that use quinolone signaling to their benefit.

## Conflicts of interest

There are no conflicts to declare.

## Supplementary Material

Supplementary information

Supplementary information

Supplementary information

Supplementary information
