# Supplementary material for: Proteome-wide mapping of PQS-interacting proteins in Pseudomonas aeruginosa
Source: Chem Sci. 2018 Jan 19;9(8):2290–4. doi: 10.1039/c7sc04287f (PMC5897874; doi:10.1039/c7sc04287f)
Supplement: Supplementary file 1 [file SC-009-C7SC04287F-s001.pdf]

Electronic Supplementary Material (ESI) for Chemical Science.
This journal is © The Royal Society of Chemistry 2018

Supporting Information

Proteome-wide mapping of PQS-interacting proteins in *Pseudomonas aeruginosa*

Rambabu Dandela,[ab] Danielle Mantin,[a] Benjamin F. Cravatt,[c] Josep Rayo[a]*
and Michael M. Meijler[a]*

[a]Department of Chemistry and National Institute for Biotechnology in the Negev, Ben-Gurion University of the Negev, PO Box 653 Beer-Sheva, 8410501 Israel

[b]Organic Chemistry Division, CSIR-National Chemical Laboratory, Dr. Homi Bhabha Road, Pune-411008, India

[c]The Skaggs Institute for Chemical Biology and Department of Chemical Physiology, The Scripps Research Institute, 10550 North Torrey Pines Road, La Jolla, CA 92037, USA

*Correspondence: pepravo@gmail.com (J.R.); meijler@bgu.ac.il (M. M. M.)

Table of Contents

General methods 2

Synthetic procedures 3

Synthesis of 3-hydroxy-2-(2-(3-(pent-4-ynyl)-3H-diazirin-3-yl)ethyl)quinolin-4(1H)-one (1)...3

Synthesis of 2-(2-(3-(pent-4-ynyl)-3*H*-diazirin-3-yl)ethyl)quinolin-4(1*H*)-one (2)....................7

Synthesis of 4-(3-(pent-4-ynyl)-3*H*-diazirin-3-yl)butan-2-one (3) 9

Synthesis of 2-heptyl-3-hydroxyquinolin-4(1*H*)-one (PQS) 9

Synthesis of 2-heptylquinolin-4(1*H*)-one (HHQ) 10

Biological evaluations 12

***E. coli* / *P. aeruginosa* pqsR agonist/competition assays** 12

Probe treatment *in vivo* and in-gel fluorescence detection 13

Enrichment for chemical proteomics LC-MS/MS 13

Extended data..15

Supplementary Tables 15

Supplementary Figures....15
References....18
NMR and MS data....21

General methods

All chemical reagents were purchased from Aldrich or Acros and used without further purification. Reactions were performed using oven-dried glassware apparatus under an atmosphere of nitrogen with anhydrous, freshly distilled solvents unless otherwise stated. THF was distilled from sodium benzophenone ketyl and DCM from CaH_2, and were applicable, solvents were freshly distilled under nitrogen immediately prior to use. N-methylpyrrolidinone (NMP) was used as commercially supplied (anhydrous/reagent grade). All other reagents were used as obtained from commercial sources. Reactions involving microwave irradiation were performed in 10 mL or 30 mL microwave tubes with clip lids using a CEM Discover® microwave apparatus (with internal temperature measurement). Thin-layer chromatography was performed using silica gel 60 with F254 indicator on glass plates (Merck). Flash chromatography was performed using Merck 40-63 µm silica gel. Solvent ratios for the purification of compounds by flash chromatography are reported as percent volume (v/v). NMR spectra were recorded using a Bruker Avance DPX400 (400 MHz) or Bruker Avance DMX500 (500 MHz) spectrometer. Spectra were calibrated on residual solvent signal. Analytical HPLC analyses were performed on a Surveyor Plus HPLC System (Thermo Scientific) using a Luna C18, 10 µm (150 x 4.6 mm) column at a flow rate of 1 mL/min. Preparative HPLC was routinely performed on a Sapphire 600 instrument (ECOM) using a Luna C18 column, 10 µm (250 x 21.20 m), at a flow rate of 20 mL/min. All runs used linear gradients from 0.1% aqueous TFA (solvent A) to 90% aqueous acetonitrile containing 0.1% TFA (solvent B). Compounds were identified by UV detection at dual wavelengths (230 nm, 260 nm). All MS analyses were performed on a LCQ Fleet mass spectrometer (Thermo Scientific) with an ESI source. High Resolution Mass Spectrometry (HRMS) data

were obtained with an Thermo Scientific Orbitrap Mass Spectrometer with an EI, ESI or APCI ion source, and reported in atomic mass units.

Synthetic procedures

Synthesis of 3-hydroxy-2-(2-(3-(pent-4-ynyl)-3H-diazirin-3-yl)ethyl)quinolin-4(1H)-one (1)

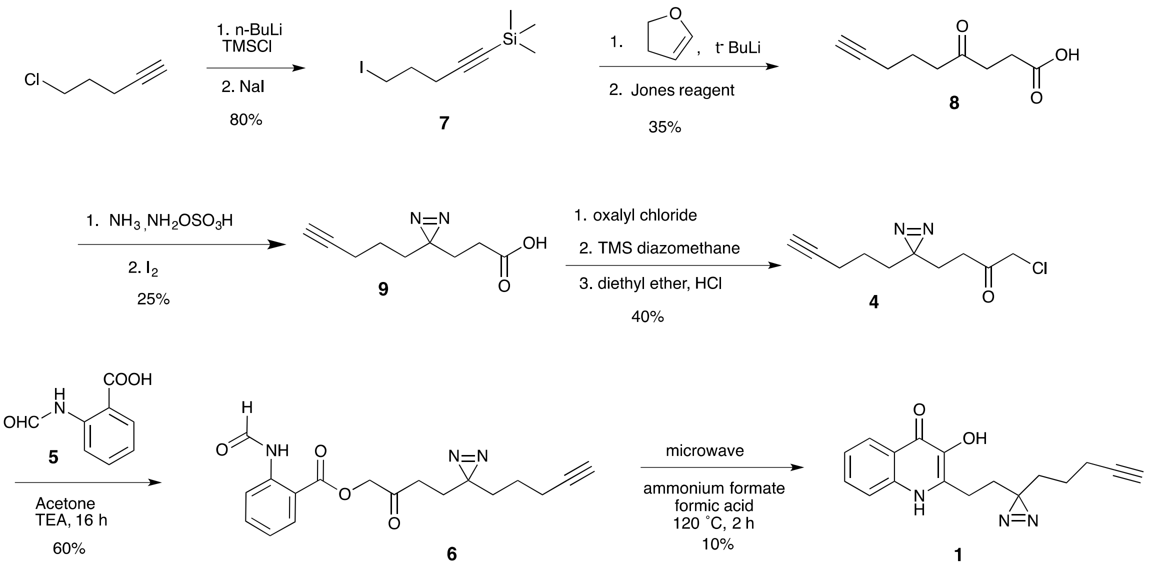

4–oxonon-8–ynoic acid (8)

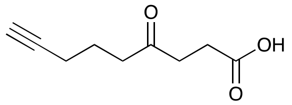

4–Oxonon-8–ynoic acid (**8**) was prepared by a known procedure[1] via lithiation of 2,3-dihydrofuran and alkylation with TMS-protected alkyne iodide **7**, followed by direct oxidation of the crude hydroxynonynone using Jones' reagent[1]. Spectral data were found to be identical to literature values.[1]

3-(3-(pent-4-ynyl)-3*H*-diazirin-3-yl)propanoic acid (9)

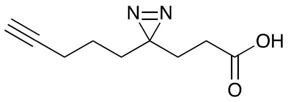

3-(3-(pent-4-ynyl)-3H-diazirin-3-yl)propanoic acid (**9**) was prepared following procedures described by Dubinsky et al.2 Anhydrous ammonia (3.5 mL) was condensed into a round bottomed flask containing 4–oxonon-8–ynoic acid (0. 50 g, 2. 9 mmol), cooled using a dry ice/acetone bath. The mixture was stirred at -35-40 °C for 5 h. The solution was cooled through addition of dry ice to the bath, and a solution of hydroxylamine-O-sulfonic acid (0.37 g, 3.3 mmol) in anhydrous methanol (1.4 mL) was added over a period of 30 minutes and the mixture was refluxed with stirring at -35 °C for 1 h. The mixture was further stirred overnight during which the excess ammonia allowed to evaporate.. The resulting slurry was filtered and the filter cake was washed with several portions of methanol. The combined filtrates were concentrated in vacuo, after which the residue was dissolved in dichloromethane (1 mL) and treated with triethylamine (0.5 mL). A solution of iodine (0.5 g, 3.9 mmol) in dichloromethane (1.7 mL) was slowly added under stirring until the appearance of a persistent orange-brown color. After concentration in vacuo the residue was chromatographed on a column of silica gel (EtOAc: DCM 20:80) to yield **9** (0.13 g, 25%) as a yellow oil. 1H NMR (400 MHz, CDCl3) δ (ppm) 2.18-2.11 (m, 4H), 1.93 (t, J = 2.66 Hz, 1H), 1.76-1.72 (m, 2H), 1.55-1.49 (m, 2H), 1.36-1.27 (m, 2H). 13C NMR (100 MHz, CDCl3) δ (ppm) 177.33, 83.11, 68.99, 31.43, 28.03, 27.84, 27.54, 22.50, 17.76. MS (ESI) m/z: [M+1] 181.1 (20%), 153.0 (100%).

1-chloro-4-(3-(pent-4-ynyl)-3*H*-diazirin-3-yl)butan-2-one (**4**)

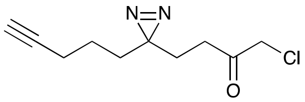

To a solution of 3-(3-(pent-4-ynyl)-3*H*-diazirin-3-yl)propanoic acid (0.23 g, 1.27 mmol) in dichloromethane (10 mL) was added oxalyl chloride (0.486 g, 3.83 mmol) and a drop of DMF. The reaction was stirred at ambient temperature for 2 h. The volatiles were evaporated, and the acid chloride was dissolved in THF (10 mL) and cooled to 0 °C. The solution was treated with a 2M solution of TMS-diazomethane in hexane (1.9 mL, 3.83

mol) over 10 min. The resulting yellow solution was allowed to stand at room temperature for 12 h. The solution was then concentrated and the residue was dissolved in THF (5 mL) and treated slowly with a 4N HCl in ether solution (5 mL). The result was allowed to stir at 24 °C for 3 h. After concentration *in vacuo* the residue was chromatographed on a column of silica gel (EtOAc: DCM 10:90) to yield **4** (0.11 g, 40%) as a yellow oil. ^{1}H NMR (400 MHz, $CDCl_3$) δ (ppm) 4.02 (s, 2H), 2.37-2.32 (m, 2H), 2.17-2.12 (m, 2H), 1.93 (t, J = 2.67 Hz, 1H), 1.76 (t, J = 7.31 Hz, 2H), 1.53-1.49 (m, 2H), 1.35-1.27 (m, 2H). ^{13}C NMR (100 MHz, $CDCl_3$) δ (ppm) 200.96, 84.03, 71.97, 49.17, 33.60, 31.38, 28.43, 26.44, 22.84, 17.51. MS (ESI) *m/z*: [M+1] 213.1 (20%), 185.0 (100%).

***N*-formyl anthranilic acid**[3] (**5**)

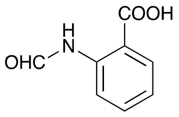

Anthranilic acid, (219.5 mg, 1.6 mmol) was stirred in formic acid (5 mL) at rt for 3 days until precipitation formed, which was collected by vacuum filtration. The solid was washed with minimum formic acid and crystallised from EtOAc/PE (40–60 °C) (7:3) to afford the title compound as a white solid (154 mg, 52%). Spectral data were found to be identical to literature values.[3]

2-oxo-4-(3-(pent-4-ynyl)-3*H*-diazirin-3-yl)butyl 2-formamidobenzoate (**6**)

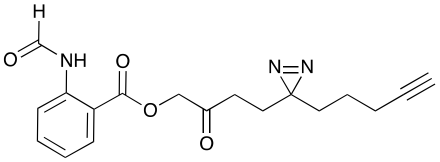

To the solution of *N*-formyl anthranilic acid (0.1 g, 0.606 mmol) in acetone (8 mL) was added triethylamine (0.074 g, 0.727 mmol) and 1-Chloro-4-(3-(pent-4-ynyl)-3*H*-diazirin-3-yl)butan-2-one (0.080 g, 0.377 mmol) and the reaction mixture was stirred at room temperature for 16 h until the reaction was complete (TLC, EtOAc). The solvent was rotary evaporated and the crude compound was washed with water and the product was used next step without further purification. ^{1}H NMR (400 MHz, DMSOd_6) δ (ppm)

10.50 (br.s, 1H), 8.42-8.40 (m, 2H), 7.98 (dd, *J* = 7.95, 1.39 Hz, 1H), 7.61 (td, *J* = 7.61, 1.22 Hz, 1H), 7.21 (t, *J* = 7.96 Hz, 1H), 5.01 (s, 2H), 2.73 (t, *J* = 2.65 Hz, 1H), 2.34 (t, *J* = 7.49 Hz, 2H), 2.09 (td, *J* = 7.00, 2.65 Hz, 2H), 1.60 (t, *J* = 7.29 Hz, 2H), 1.45-1.39 (m, 2H), 1.23-1.15 (m, 2H). ^{13}C NMR (100 MHz, DMSOd_6) δ (ppm) 207.52, 170.89, 166.14, 144.21, 139.65, 135.99, 128.64, 126.38, 121.42, 88.81, 76.77, 73.63, 37.28, 36.11, 33.23, 30.80, 27.59, 22.26. MS (ESI) *m/z*: [M+1] 342.1 (20%), 314.0 (100%).

3-hydroxy-2-(2-(3-(pent-4-ynyl)-3*H*-diazirin-3-yl)ethyl)quinolin-4(1*H*)-one (**1**)

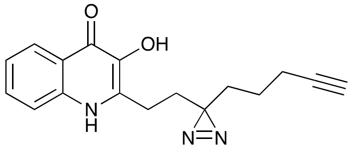

The appropriate 2-oxo-4-(3-(pent-4-ynyl)-3*H*-diazirin-3-yl)butyl 2-formamidobenzoate (0.1 g, 0.29 mmol), ammonium formate (0.184 g, 2.93 mmol) was dissolved in excess formic acid (4.2 mL, 0.61 M) and transferred to microwave vials. In batches, the mixture was subjected to microwave irradiation (300w, 150psi) at 120 °C for 60 min until the starting material was consumed (TLC, EtOAc). To each vial, H_2O was added and the mixtures were combined and extracted three times with EtOAc. The organic phase was washed with brine and dried over $MgSO_4$. After concentration *in vacuo* the residue was purified by HPLC to afford **7**. yield (8.6 mg, 10%). ^{1}H NMR (400 MHz, DMSOd_6) δ (ppm) 11.48 (br. s, 1H), 8.03 (d, *J* =7.31 Hz,1H), 7.53-7.45 (m, 2H), 7.21-7.17 (m, 1H), 2.73 (t, *J* = 2.62 Hz, 1H), 2.56-2.52 (m, 2H), 2.09 (dd, *J* = 7.21, 2.78 Hz, 2H), 1.71 (t, *J* = 8.11 Hz, 2H), 1.49 (t, *J* = 8.20 Hz, 2H), 1.25-1.17 (m, 2H). ^{13}C NMR (100 MHz, DMSOd_6) δ (ppm) 169.17, 138.30, 137.85, 134.53, 130.70, 125.00, 122.69, 122.24, 118.31, 84.14, 72.19, 31.38, 31.21, 28.97, 23.45, 23.04, 17.68. MS (ESI) *m/z*: [M+1] 296.0 (10%), 268.2 (100%). HRMS (ESI) *m/z* calcd for $C_{17}H_{17}N_3O_2H^+$ (M+1) 296.13935, found 296.14008.

Synthesis of 2-(2-(3-(pent-4-ynyl)-3*H*-diazirin-3-yl)ethyl)quinolin-4(1*H*)-one (2)

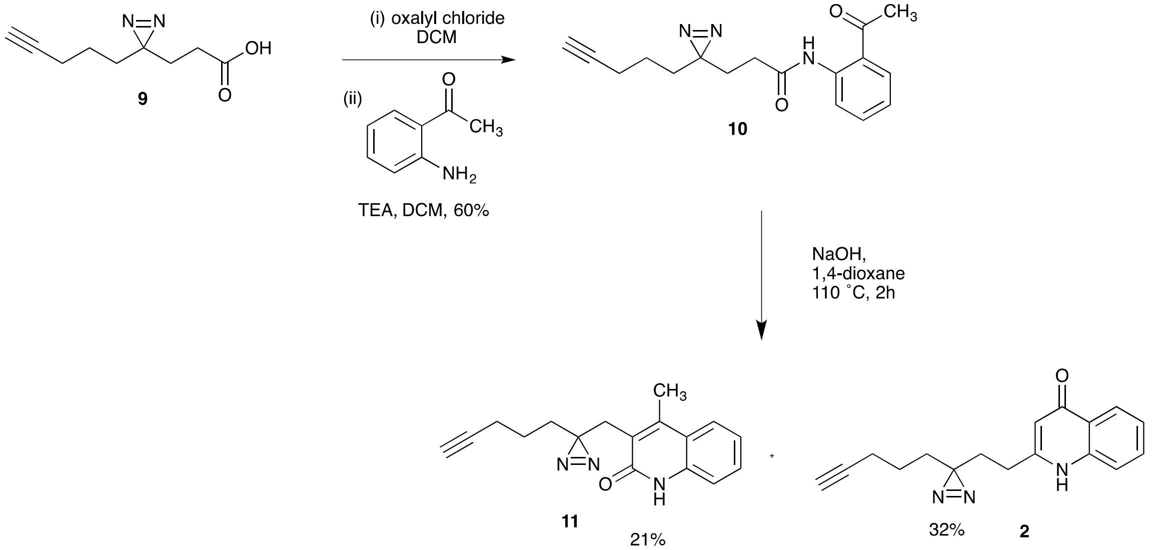

N-(2-acetylphenyl)-3-(3-(pent-4-ynyl)-3*H*-diazirin-3-yl)propanamide (10)

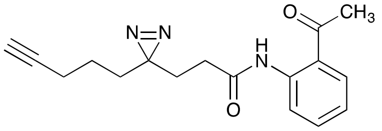

Step 1. To a solution of 3-(3-(pent-4-ynyl)-3*H*-diazirin-3-yl)propanoic acid (0.20 g, 1.1 mmol) in dichloromethane (10 mL) was added oxalyl chloride (0.423 g, 3.3 mmol) and a drop of DMF. The reaction was stirred at ambient temperature for 2 h. The volatiles were evaporated, and the acid chloride was used for next step.

Step 2. To a solution of amino acetophenone (0.22 g, 1.66 mmol) in CH_2Cl_2 (10 mL) was added triethylamine (0.33 g, 3.3 mmol). The mixture was cooled to 0-5 °C followed by the addition of 3-(3-(pent-4-ynyl)-3*H*-diazirin-3-yl)propanoic acid chloride (from step 1) and stirred for 2 h at room temperature. 0.1 N HCl (20 mL) was added to the reaction mixture and extracted with CH_2Cl_2 (3 x 20 mL). The combined organic layers were washed with saturated aqueous $NaHCO_3$ (30 mL), and brine (30 mL). The organic layer was dried over Na_2SO_4 and concentrated *in vacuo*. The resultant crude residue was used for next without purification. ^{1}H NMR (400 MHz, $CDCl_3$) δ (ppm) 11.69 (br.s, 1H), 8.68 (d, *J* =7.3 Hz,1H), 7.85 (dd, *J* = 8.07, 1.21 Hz, 1H), 7.52 (td, *J* = 7.96, 1.22 Hz, 1H), 7.11-7.06 (m, 1H), 2.63 (s, 3H), 2.20 (t, *J* = 7.6 Hz, 2H), 2.15-2.11 (m, 2H), 1.91-1.90 (m, 1H), 1.84 (t,

J = 7.96 Hz, 2H), 1.56-1.52 (m, 2H), 1.35-1.28 (m, 2H). ^{13}C NMR (100 MHz, $CDCl_3$) δ (ppm) 202.93, 170.61, 140.85, 135.26, 131.72, 122.49, 121.75, 120.79, 83.33, 69.14, 32.43, 31.69, 28.61, 28.45, 27.94, 22.70, 17.92. MS (ESI) *m/z*: [M+1] 298.1 (20%), 270.1 (100%).

2-(2-(3-(pent-4-ynyl)-3*H*-diazirin-3-yl)ethyl)quinolin-4(1*H*)-one (2)

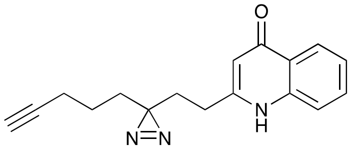

To a solution of *N*-(2-acetylphenyl)-3-(3-(pent-4-ynyl)-3*H*-diazirin-3-yl)propanamide **10** (0.1 g, 0.33 mmol) in 1,4-dioxane (8.0 mL) was added sodium hydroxide (41 mg, 1.01 mmol) and the resulting solution was heated to 110 °C under argon for 3 hours. After cooling to room temperature, all volatile components were then removed under reduced pressure and the crude residue was purified by column chromatography to afford **2** in 32% yield (30 mg) and **11** in 21% yield (19.8 mg).

^{1}H NMR (400 MHz, $CDCl_3$) δ (ppm) 8.35 (dd, *J* = 8.2, 1.4 Hz, 1H), 7.63 (td, *J* = 7.3, 1.5 Hz, 1H), 7.44 (d, *J* = 8.3 Hz, 1H), 7.38 (t, *J* = 7.4 Hz, 1H), 6.20 (s, 1H), 2.45 (t, *J* = 7.8 Hz, 2H), 2.14 (td, *J* = 6.9, 2.4 Hz, 2H), 1.94 (t, *J* = 2.4 Hz, 1H), 1.92-1.88 (m, 2H), 1.56 (t, *J* = 8.0 Hz, 2H), 1.34-1.26 (m, 2H). ^{13}C NMR (100 MHz, $CDCl_3$) δ (ppm) 178.77, 151.25, 139.76, 132.21, 126.01, 125.02, 123.99, 117.36, 108.85, 83.08, 69.32, 32.25, 31.45, 28.57, 27.80, 22.51, 17.78. MS (ESI) *m/z*: [M+1] 279.9 (18%), 252.1 (100%). HRMS (ESI) *m/z* calcd for $C_{17}H_{17}N_3OH^+$ (M+1) 280.14444, found 280.14456.

4-methyl-3-((3-(pent-4-yn-1-yl)-3H-diazirin-3-yl)methyl)quinolin-2(1*H*)-one (11)

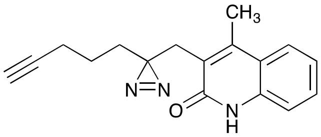

^{1}H NMR (400 MHz, $CDCl_3$) δ (ppm) 7.32 (d, *J* = 8.11 Hz, 1H), 7.56-7.49 (m, 2H), 7.27-7.23 (m, 1H), 3.08 (s, 2H), 2.44 (s, 3H), 2.25 (td, *J* = 6.81, 2.38 Hz, 2H), 1.94 (t, *J* = 2.65 Hz, 1H), 1.74-1.70 (m, 2H), 1.53-1.46 (m, 2H). ^{13}C NMR (100 MHz, $CDCl_3$) δ (ppm) 163.68, 146.34,

137.34, 130.05, 124.53, 124.17, 122.41, 120.50, 116.42, 83.59, 68.91, 32.24, 29.88, 28.88, 22.88, 17.99, 16.08. MS (ESI) *m/z*: [M+1] 280.0 (100%).

Synthesis of 4-(3-(pent-4-ynyl)-3*H*-diazirin-3-yl)butan-2-one (3)

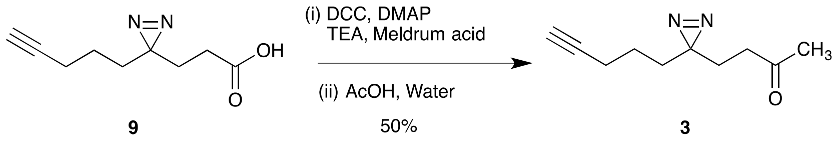

Dicyclohexyl carbodiimide (57 mg, 0.277 mmol), *N,N'*-dimethyl-4-aminopyridine (6.7 mg, 0.05 mmol) and triethylamine (0.04 mL, 0.277 mmol) were added to a solution of Meldrum's acid (40 mg, 0.277 mmol) in dichloromethane (5 ml). To the reaction mixture 3-(3-(Pent-4-ynyl)-3*H*-diazirin-3-yl)propanoic acid (**9**) (50 mg, 0.277 mmol) was added and the reaction mixture was stirred overnight. The reaction mixture was filtered and the solid was washed with dichloromethane (2 x 5 mL). The filtrate and washings were combined and evaporated under reduced pressure to give an orange oil. Acetic acid (3 mL) and water (3 mL) were added and the reaction was heated at 90 ^{o}C for 4 hours. After the solution had cooled to room temperature, it was extracted with ethyl acetate (15 mL). The organic extract was dried ($MgSO_4$) and evaporated to give a residue that was purified by column chromatography on silica gel (EtOAc: Hexane 6:94) to yield (24 mg, 50 %) as a colorless oil. ^{1}H NMR (400 MHz, $CDCl_3$) δ (ppm) 2.24-2.17 (m, 4H), 2.14 (s, 3H), 1.98 (t, *J* = 2.29 Hz, 1H), 1.74 (t, *J* = 7.32 Hz, 2H), 1.56-1.52 (m, 2H), 1.40-1.33 (m, 2H). ^{13}C NMR (100 MHz, $CDCl_3$) δ (ppm) 206.66, 83.31, 69.00, 37.22, 31.88, 29.90, 27.85, 26.54, 22.66, 17.88. MS (ESI) *m/z*: [M+1] 179.1 (10%), 151.0 (100%).

Synthesis of 2-heptyl-3-hydroxyquinolin-4(1*H*)-one (PQS)

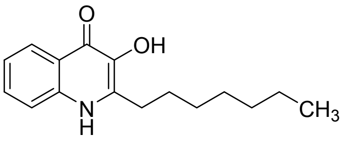

2-heptyl-3-hydroxyquinolin-4(1*H*)-one (**PQS**) was prepared by a known procedure[5] Spectral data were found to be identical to literature values.[5,6]

^{1}H (400 MHz, d_6-DMSO) δ (ppm) 11.61-11.18 (m, 1H), 8.04 (d, *J* = 8.19 Hz, 1H), 7.49 (d, *J* = 3.16 Hz, 2H), 7.19-7.14 (m, 1H), 2.72-2.65 (m, 2H), 1.65-1.58 (m, 2H), 1.33-1.17 (m, 8H), 0.85-0.78 (m, 3H). ^{13}C (100 MHz; d_6-DMSO) δ (ppm) 169.34, 138.30, 137.85, 135.97, 130.43, 124.96, 122.66, 121.97, 118.25, 31.68, 29.27, 28.95, 28.61, 28.29, 22.54, 14.42; MS (ESI) *m/z*: [M+1] 260.0 (100%). HRMS (ESI) m/z calcd for $C_{16}H_{21}NO_2H^+$ (M+1) 260.16451, found 260.16479.

Synthesis of 2-heptylquinolin-4(1*H*)-one (HHQ)

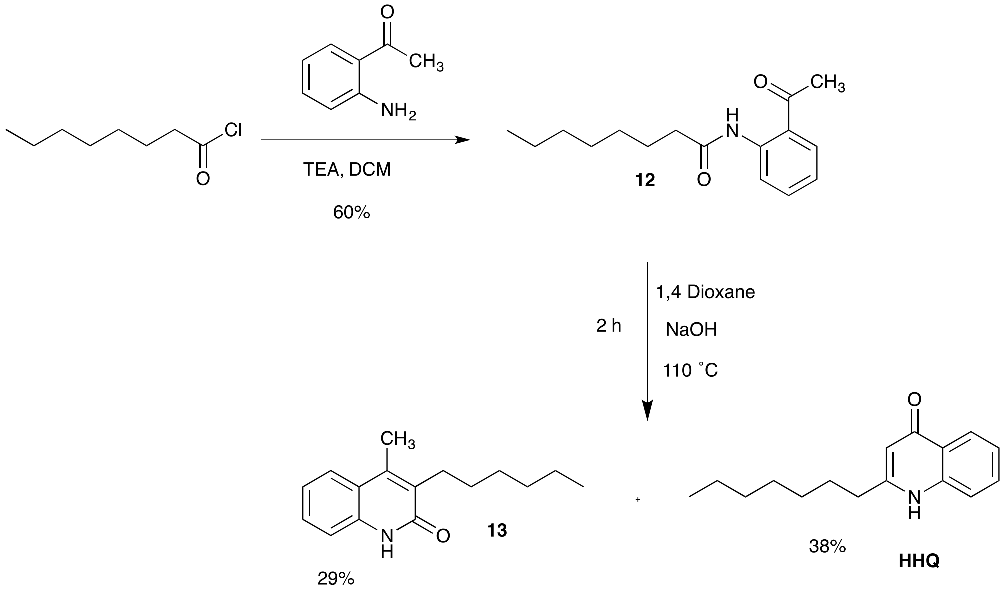

Synthesis of *N*-(2-acetylphenyl)octanamide (12)[4]

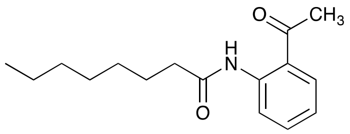

To a solution of amino acetophenone (1.00 g, 7.40 mmol) in CH_2Cl_2 (100 mL) was added Et_3N (3.2 mL 22.2 mmol), followed by the addition of octanoyl chloride (2.52 mL, 14.8 mmol) at 0 ^{o}C and the reaction mixture was stirred for 2 h at room temperature. 0.1 N HCl (5 mL) was added to the reaction mixture and extracted with CH_2Cl_2 (80 mL). The

combined organic layers were washed with saturated aqueous $NaHCO_3$ (15 mL), and brine (15 mL). The organic layer was dried over Na_2SO_4 and concentrated *in vacuo*. The resultant crude residue was purified using column chromatography to afford **12**. Yield: 1.15 g (60%); ^{1}H NMR(400 MHz, $CDCl_3$) δ (ppm) 11.68 (s, 1H), 8.78-8.72 (m, 1H), 7.87 (dd, *J* = 8.02, 1.56 Hz, 1H), 7.54-7.50 (m, 1H), 7.10-7.08 (m, 1H), 2.64 (s, 3H), 2.45 (t, *J* = 7.2 Hz, 2H), 1.76-1.68 (m, 2H), 1.39-1.22 (m, 8H), 0.85 (t, *J* = 6.88 Hz, 3H). ^{13}C NMR (100 MHz,$CDCl_3$) δ (ppm) 202.72, 172.74, 141.07, 135.07, 131.51, 122.05, 121.58, 120.67, 38.72, 31.59, 29.09, 28.90, 28.52, 25.40, 22.51, 13.97. MS (ESI) *m/z*: [M+1] 262.0 (100%).

Synthesis of 2-heptylquinolin-4(1*H*)-one (HHQ)

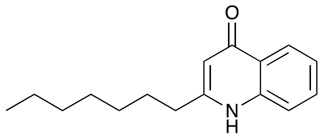

To a solution of *N*-(2-acetylphenyl)octanamide **14** (200 mg, 0.76 mmol) in 1,4-dioxane (8.0 mL) was added sodium hydroxide (92 mg, 2.29 mmol) and the resulting solution was heated to 110 °C under argon for 3 hours. After cooling to r.t., all volatile components were then removed under reduced pressure and the crude residue was purified by column chromatography to afford **15** in 38% yield (70 mg) and **16** in 29% yield (54 mg). Spectral data were found to be identical to literature values of compound **15**.[6]

^{1}H NMR(400 MHz, $CDCl_3$) δ (ppm) 8.35 (d, *J* = 8.22 Hz, 1H), 7.84 (d, *J* = 8.31 Hz, 1H), 7.56 (t, *J* = 7.67 Hz, 1H), 7.30 (t, *J* = 7.49 Hz, 1H), 6.24 (s, 1H), 2.69 (t, *J* = 7.74 Hz, 2H), 1.77-1.62 (m, 2H), 1.30-1.05 (m, 8H), 0.77 (t, *J* = 7.39 Hz, 3H). ^{13}C NMR (150 MHz, $CDCl_3$) δ (ppm) 178.78, 155.74, 140.80, 131.63, 124.96, 124.86, 123.45, 118.77, 107.87, 34.28, 31.56, 29.13, 28.88, 22.47, 13.93. MS (ESI) *m/z*: [M+1] 244.2 (100%). HRMS (ESI) *m/z* calcd for $C_{16}H_{21}NOH^+$ (M+1) 244.16959, found 244.16992.

3-hexyl-4-methylquinolin-2(1*H*)-one (13)

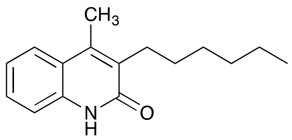

^{1}H NMR(400 MHz, $CDCl_3$) δ ppm) 7.69 (d, *J* = 8.00 Hz, 1H), 7.48-7.45 (m, 2H), 7.24-7.19 (m, 1H), 2.85 (t, *J* = 7.86 Hz, 2H), 2.51 (s, 3H), 1.64-1.56 (m, 2H), 1.53-1.46 (m, 2H), 1.42-1.32 (m, 4H), 0.94 (t, *J* = 7.15 Hz, 3H). ^{13}C NMR (100 MHz, $CDCl_3$) δ (ppm) 164.18, 143.05, 136.91, 131.67, 129.06, 124.16, 122.10, 121.08, 116.27, 31.84, 29.59, 29.09, 27.01, 22.72, 15.09, 14.23. MS (ESI) *m/z*: [M+1] 244.3 (100%).

Biological evaluations

E. coli DH5α *pqsR* agonist assay

E. coli DH5α harboring the PqsR expression vector, pEAL08-2, and a plasmid-borne pqsA-lacZ fusion was used to quantify quorum sensing activation by measuring expression levels of β-galactosidase.[7] Bacteria were incubated overnight in LB medium containing 100 μg/mL of ampicillin and 15 μg/mL of gentamicin. The culture was diluted at a 1:10 ratio by volume with fresh medium and further incubated until an OD600 of 0.3 was reached. A 96 well microtiter plate (Greiner) was prepared with the desired concentrations of inhibitors and bacteria were added to reach a final optical density at 600 nm (OD600) of 0.3. Expression was induced by the addition of L-(+)-arabinose (4 mg/mL) and the plates were incubated at 37°C for a period of 4 hours (OD600 of 0.45-0.5). The cultures were then assayed for β-galactosidase activity according to the Miller assay method[8]: 200 μL aliquots were transferred to clear 96-well microtiter plates and the OD600 was recorded. 100 μL of each well was then added to a polypropylene-based 96-well microtiter plate containing 200 μL Z-Buffer, 10 μL chloroform and 5 μL of 0.1% SDS (w/v). Wells were thoroughly rinsed by pipetting, after which the chloroform was allowed to settle. 100 μL of the aqueous upper layer was transferred to a fresh 96-well microtiter plate and 20 μL of ortho-nitrophenyl-β-D-galactopyranoside (ONPG, 4 mg/mL in phosphate buffer, pH 7) were added. The plates were incubated for 35 minutes at 28°C. The reaction was terminated by the addition of 80 μL 1 M aqueous sodium

carbonate solution, and absorption at two wavelengths (550 nm, 429 nm) was recorded. Miller units were calculated using standard methods.[8]

PAO1 Δ*PqsAH* CTX::pqsA'-lux antagonist\agonist assays

PAO1 Δ*PqsAH* CTX::pqsA'-lux was used to quantify PqsR-based quorum sensing activation by luminescence. Bacteria were incubated overnight in LB medium containing 125 μg/mL of tetracycline until an OD_{600} of 0.7 was reached. This overnight culture was diluted to an OD_{600} of 0.05 by fresh LB medium. A 96 well microtiter plate (Greiner) was prepared with the desired concentrations of PQS/HHQ probe. 100 µL of the diluted bacteria were added to each well in the presence of 110 nM of 2-heptyl-3-hydroxyquinolin-4(1H)-one (PQS) to test antagonistic activities and in the absence of PQS to test agonistic activities. Luminescence and OD_{600} values were measured using a Microtiter Plate Reader (Varioskan Flash, Thermo). The values were taken every 20 min for 14 h with continuous shaking at 37°C. The luminescence values were divided by OD_{600} values and plotted against the added compound concentration.

Probe treatment *in vivo* and in-gel fluorescence detection

PA01 was inoculated into 10 mL of LB media. Cells were grown at 37°C with shaking to an optical density (OD600) of 1.5. Cells were washed twice with fresh LB medium, and resuspended in fresh LB supplemented with 300 µM of probe (100x stock solution in DMSO) or vehicle control. After 1 h incubation at 37°C, cultures were transferred to 10 cm dishes and irradiated for 10 min with 354 nm UV light, at 9 °C. Bacteria were collected, washed twice with ice-cold PBS, and the collected pellet was stored at -20°C. Cells were resuspended in ice cold PBS, and the suspension was sonicated 6x15 s (1 min rest), 50% intensity. The samples were then centrifuged at 15 kg (30 min, 4°C), and the supernatants were collected. Protein concentration were measured using BCA assay (Pierce) and adjusted to 1 mg/ml. Proteome labelling experiments were performed using 50 µM TAMRA-azide, 1 mM TCEP, 0.1 mM TBTA, and 1 mM $CuSO_4$. The reactions were left 1 h at RT, quenched with 4X Laemmli sample buffer, resolved on 10X SDS-

PAGE, and the fluorescence for each one of the samples was recorded with a LAS-4000 (GE Healthcare) digital imaging system.

Enrichment for chemical proteomics LC-MS/MS

One milligram of pretreated PA01 lysate obtained as described above was prepared for proteomic analysis. CuACC chemistry was performed as described above, using biotin azide instead of rhodamine azide. Excess reagents were removed using methanol/chloroform precipitation: the samples were transferred to 15 mL conical tubes on ice, and 2 mL of cold MeOH and 0.5 mL of cold $CHCl_3$ were added, and the samples were vortexed. Next, 1.0 mL of cold PBS was added, the samples were vortexed, and centrifuged at 5,000 rpm for 10 min. The top and bottom layers were removed, leaving the protein interface, and the samples were washed with 1:1 MeOH:CHCl 3 (1.0 mL, 3x). 2 mL of MeOH was added and the samples were sonicated with a probe sonicator, and then 0.5 mL of $CHCl_3$ was added. Lastly, samples were centrifuged at 5,000 rpm for 10 min to pellet protein, and supernatants were removed. Next, the samples were denatured and reduced: 500 μL 6M urea (in PBS, made fresh) and 50 μL premixed 100 mM TCEP/300 mM Na_2CO_3 solution (in PBS) were added, the sample sonicated, and incubated for 30 min at 37 °C in shaker. Next, 70 uL of 400 mM iodoacetic acid was added and incubated for 30 min at room temp. In the dark, 140 uL of 10% SDS in PBS was added, and the sample diluted with 5.5 mL PBS. Enrichment: Sample was added to 100 μL of Streptavidin beads (beads were washed 3x with 500 μL PBS), and incubated 1.5 h at RT on shaker. The sample was centrifuged to pellet beads, and the beads were washed: 3x each of 10 mL 0.2% SDS/PBS, PBS, and DDW. Lastly, the beads were transferred to low-binding Eppendorf tubes using 2x 0.5 mL DDW, spun down, and the supernatant removed. Trypsinization: The following reagents were premixed and then added to each tube: 200 uL 2M urea/PBS, 2 uL 100 mM $CaCl_2$, 4 μL trypsin (20 ug reconstituted in 40 μL trypsin buffer). The tubes were shaken overnight (up to 12 h) at 37 °C. The beads were then briefly centrifuged to pellet and the supernatant and beads transferred to a small spin column, and the supernatant

collected by centrifugation. The beads were washed with 100 µL of PBS and collected in the same tube. The filtrate was acidified using 16 µL formic acid and stored at -20 °C. Samples were analyzed using an LTQ-Orbitrap XL ETD (Thermo Fisher Scientific) coupled to a NanoLC-2D (Eksigent), and proteins identified with MaxQuant version 1.5.3.30, using the Uniprot PAO1 reference proteome. Cysteine carbamidomethylation was used as a fixed modification, and methionine oxidation. Additionally, at least two peptides identification were required per protein, and the FDR was set to 0.01 for peptides and proteins.

Extended data

Supplementary Tables

See separate Excel files for the following:
Table S1. Raw data
Table S2. Probe 1 chemical proteomics
Table S3. Probe 2 chemical proteomics

Supplementary Figures

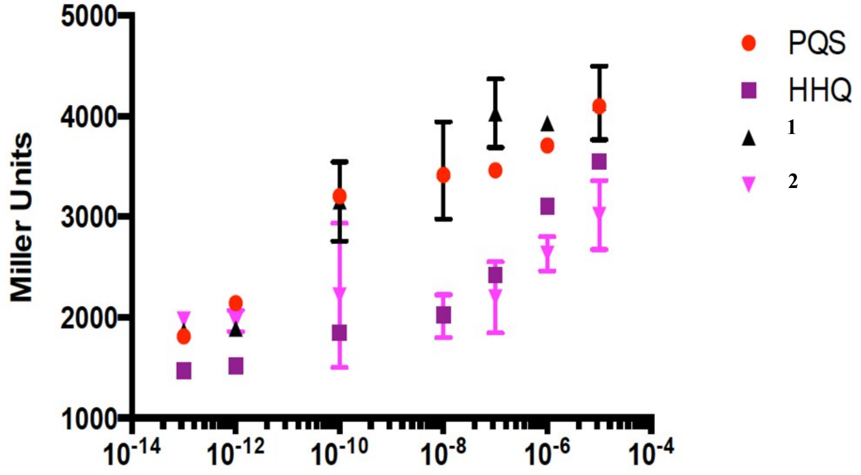

Fig S1. Quinolone probes are PqsR agonists with relative potencies that are comparable to their corresponding parent molecules. Agonist assays were performed using an *E. coli* DH5α/ pEAL08−2 (pqsA-lacZ) β-galactosidase-based PqsR reporter strain.

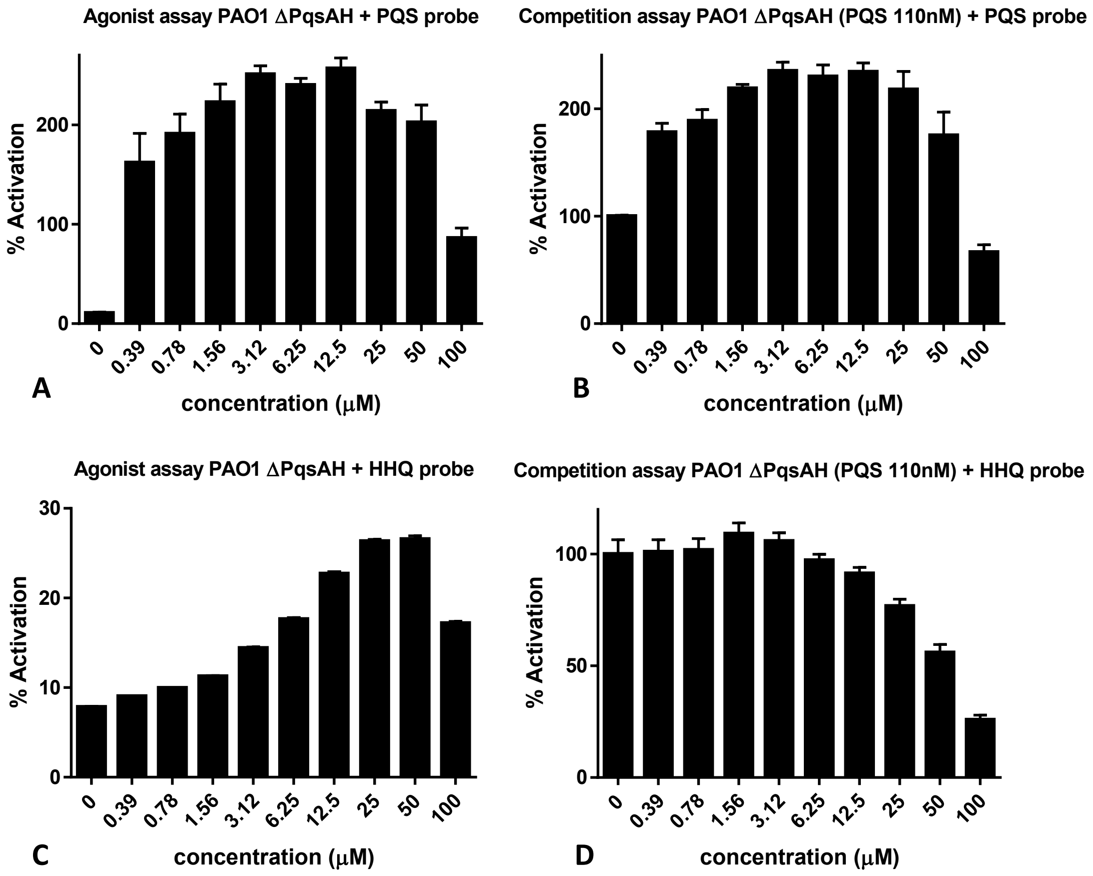

Fig S2. Both quinolone probes are PqsR agonists with markedly different potencies when tested using a *P. aeruginosa* strain that is not able to synthesize PQS and HHQ (Δ*pqsAH*): A) PQS probe **1** agonist assay; B) PQS probe **1** competition assay with 110 nM PQS; C) HHQ probe **2** agonist assay; D) HHQ probe **2** competition assay with 110 nM PQS. PQS probe **1** is a strong PqsR agonist in this reporter strain, while HHQ probe **2** is a moderate PQS competitor.

Fig S3. Labeling experiments with probe **1** (5 μM) and in competition with PQS (100 μM) in mutant strains Δ*pqsAH* (left panels) and Δ*pqsAHR* (lacking PqsR) show distinct labeling patterns and show a band with an approximate MW of 37 kDa (left panels, bottom row shows enhanced contrast) that is sharply reduced (or absent) in the right panel. The arrow indicates the probable PqsR band.

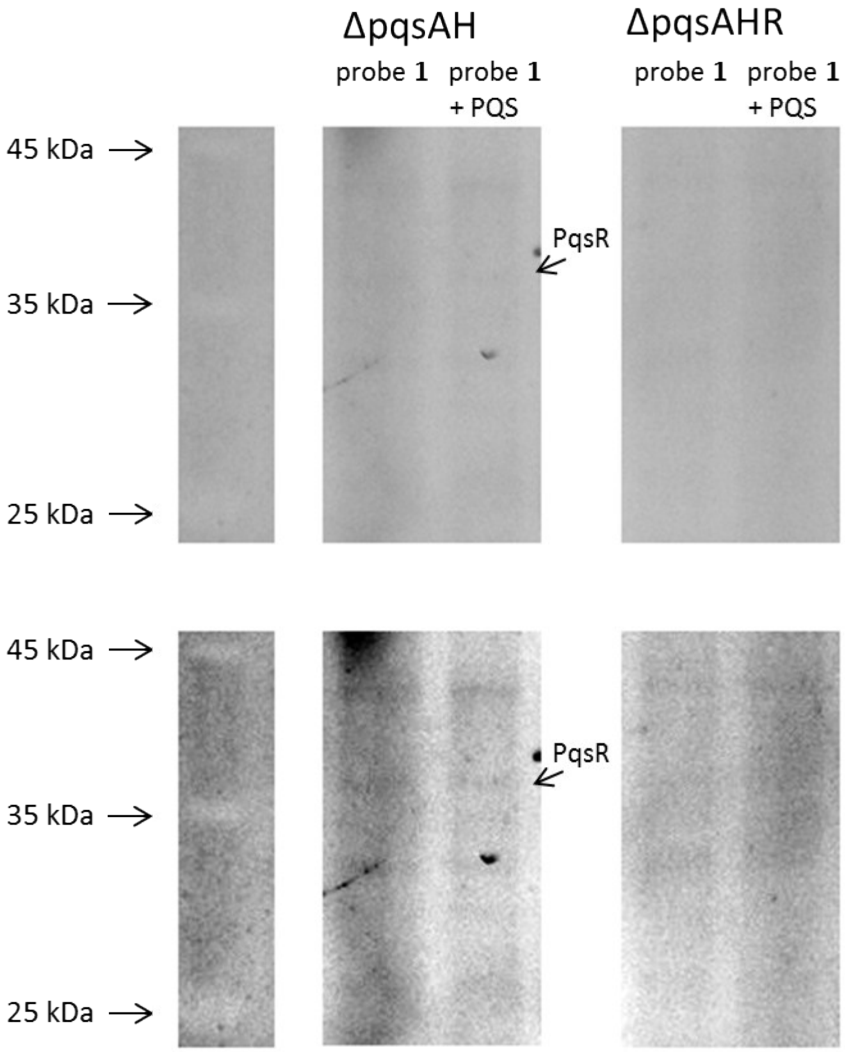

References

1. D. M. Hodgson, A. H. Labande, F. Y. Pierard, M. A. E. Castro, *J. Org. Chem.* 2003**,** **68**, 6153-6159.
2. L. Dubinsky, A. Delago, N. Amara, P. Krief, J. Rayo, T. Zor, V. V. Kravchenko, M. M. Meijler, *Chem. Commun.*, 2013, ***49***, 5826-5828.
3. W. Szczepankiewicz, J. Suwinski, *Chemistry of Heterocyclic Compounds.* 2000, ***36***, 809-810.
4. (i) C. P. Jones, K. W. Anderson, and S. L. Buchwald. *J. Org. Chem.* 2007, ***72***, 7968-7973 (ii) A. L. Searles, H. G. Lindwall, *J. Am. Chem. Soc.* 1946, ***68***, 988-990. (iii) T. G. Back, M. Parvez, J. E. Wulff, *J. Org. Chem*. 2003, ***68***, 2223-2233.
5. J. T. Hodgkinson, W. R. J. D. Galloway, S. Saraf, I. R. Baxendale, S. V. Ley, M. Ladlow, M. Welch and D. R. Spring. *Org. Biomol. Chem.***,** 2011, ***9***, 57-61.
6. D. Szamosvari, V. F. Reichle, M. Jureschi and T. Bottcher. *Chem. Commun.,* 2016, ***52***, 13440-13443.
7. C. Cugini, M. W. Calfee, J. M. Farrow, D. K. Morales, E. C. Pesci and D. A. Hogan, *Mol. Microbiol.*, 2007, **65**, 896–906.
8. Miller, J. H.; Cold Spring Harbor Laboratories: **1972**, 352.

NMR and MS data

^{1}H NMR for compound **1**:

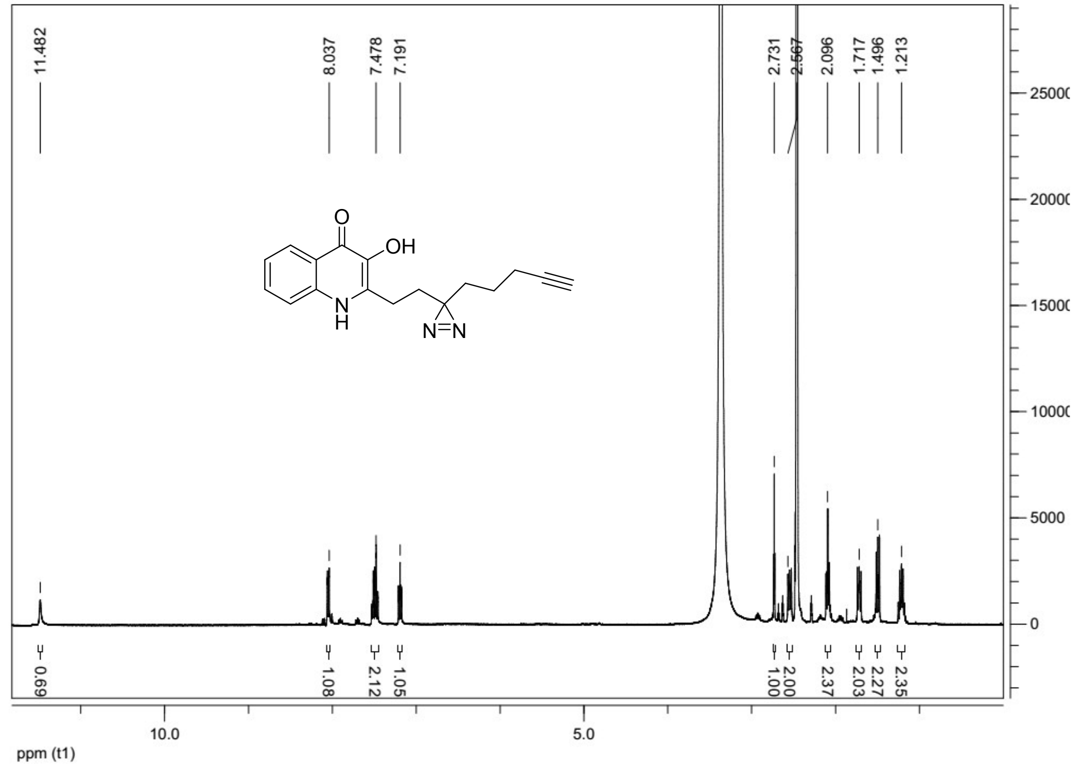

^{13}C NMR for compound **1**:

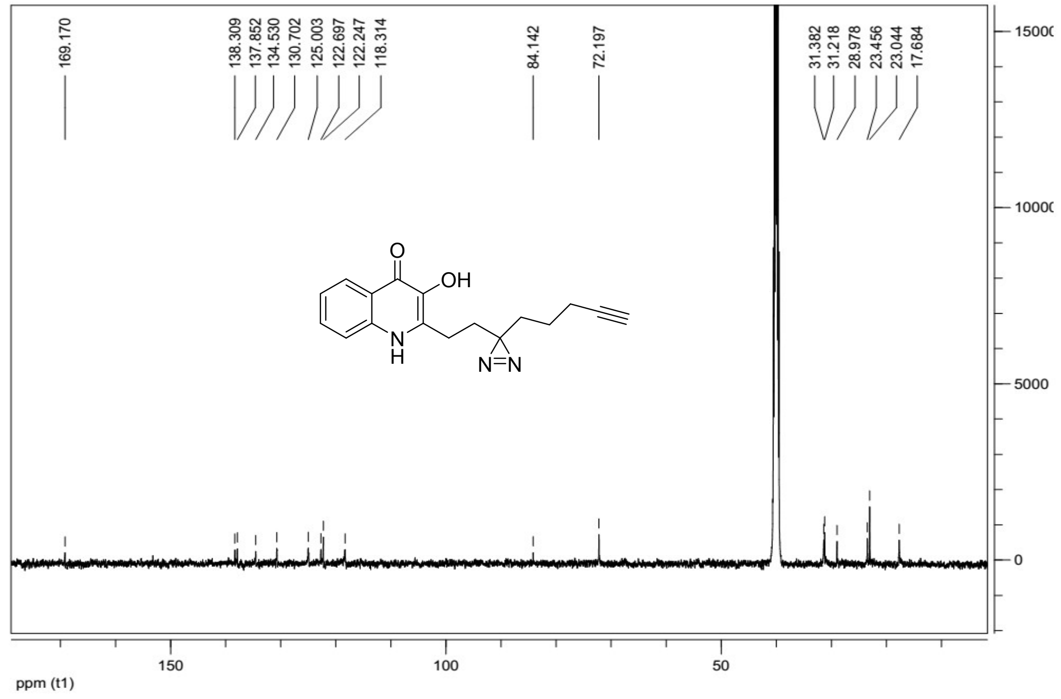

^{1}H NMR for compound **2**:

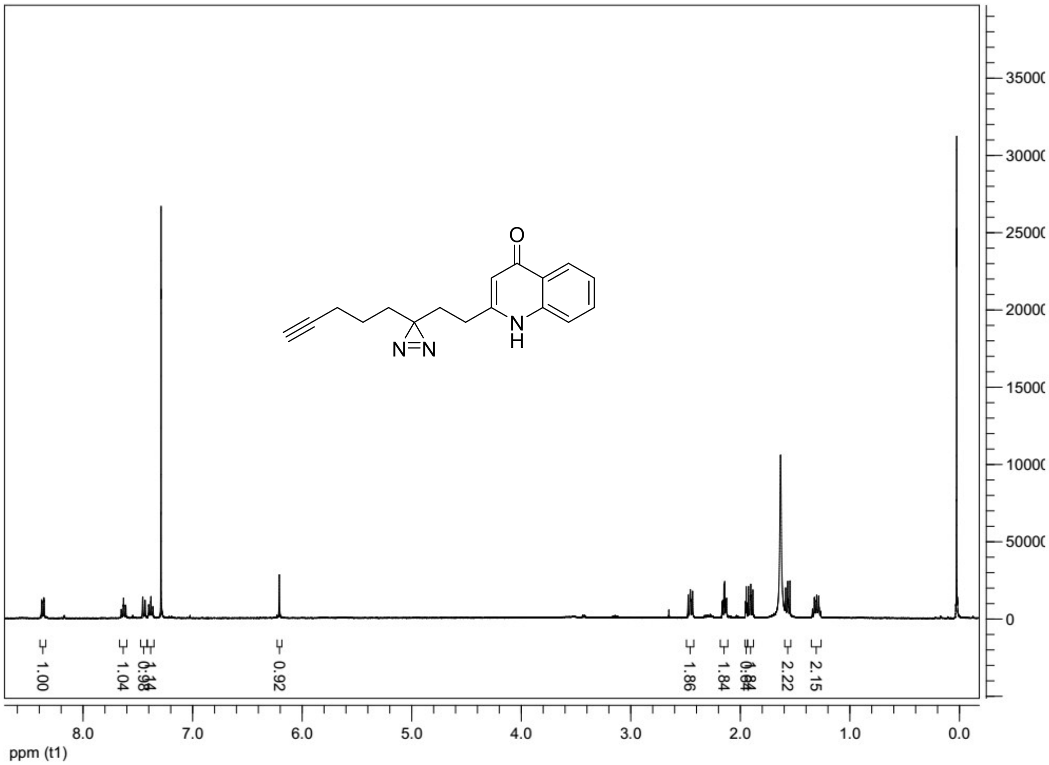

^{13}C NMR for compound **2**:

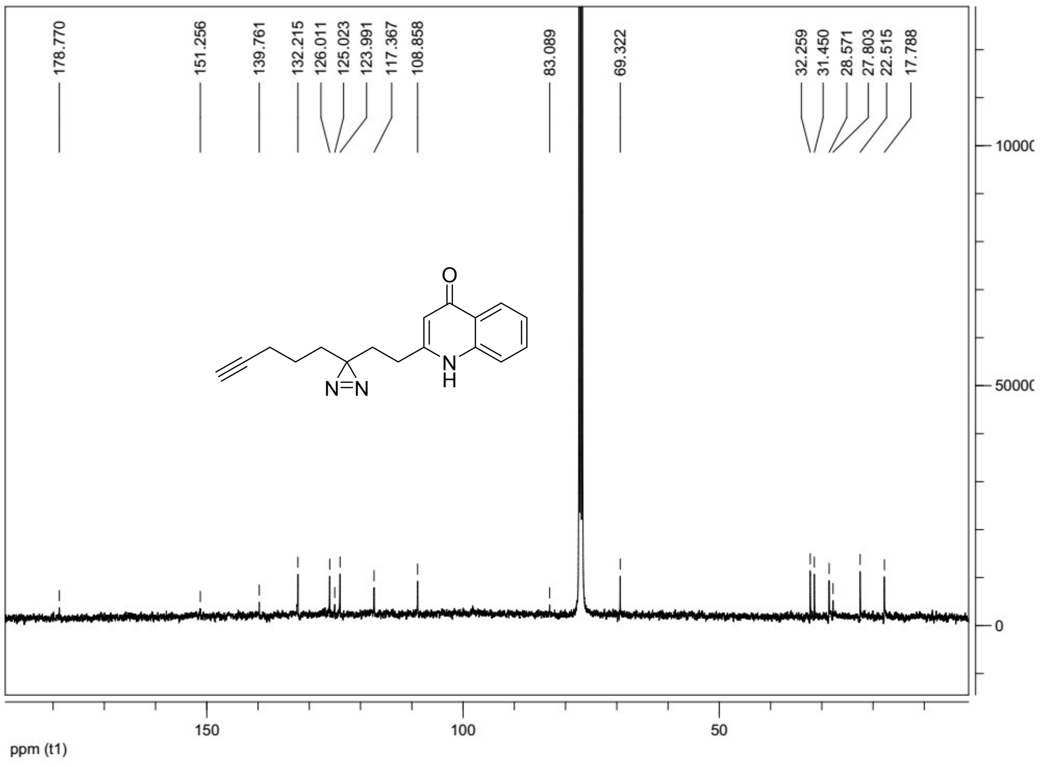

^{1}H NMR for compound **3**:

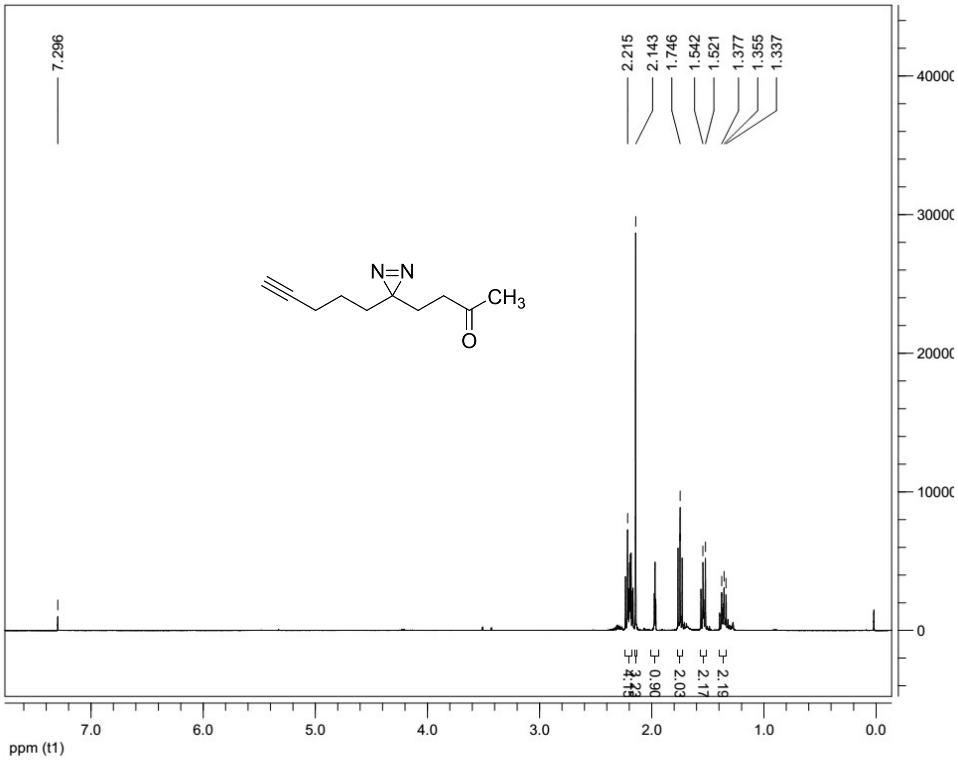

^{13}C NMR for compound **3**:

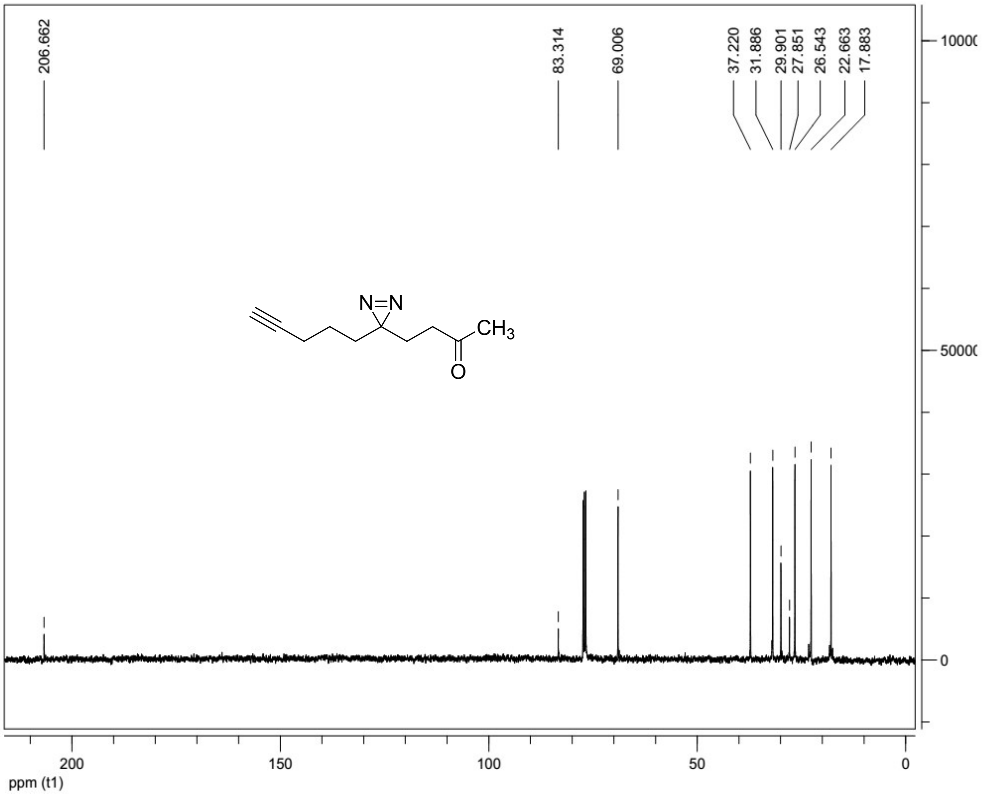

^{1}H NMR for compound **4**:

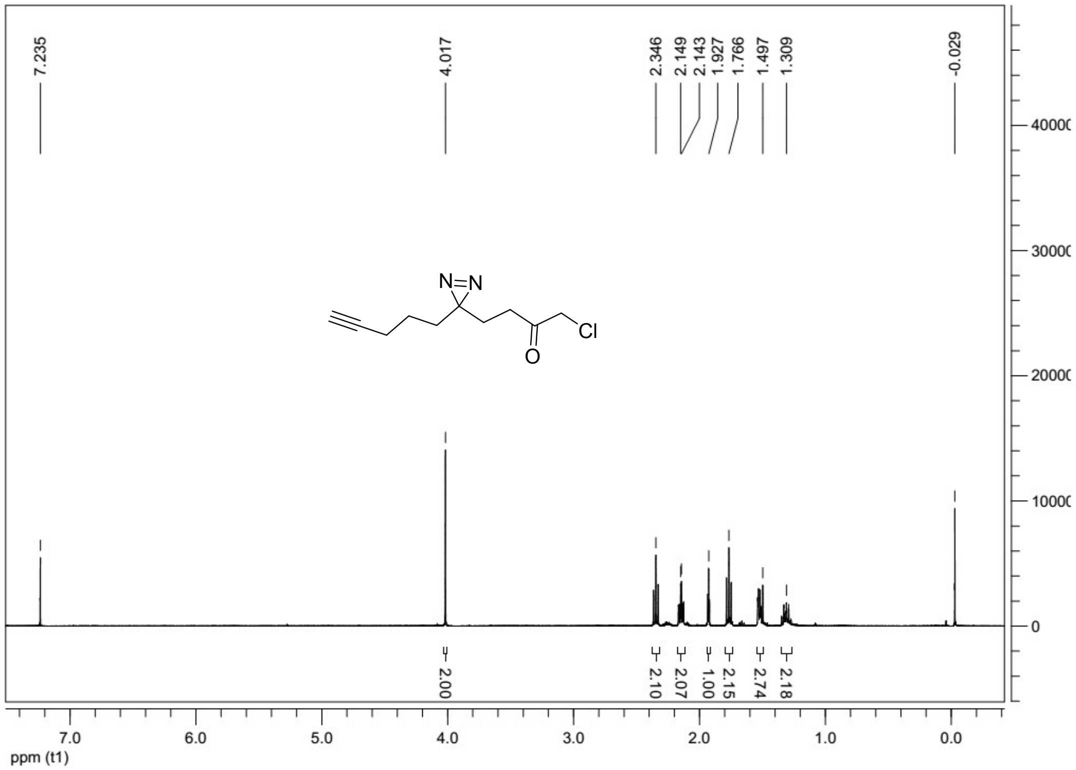

^{13}C NMR for compound **4**:

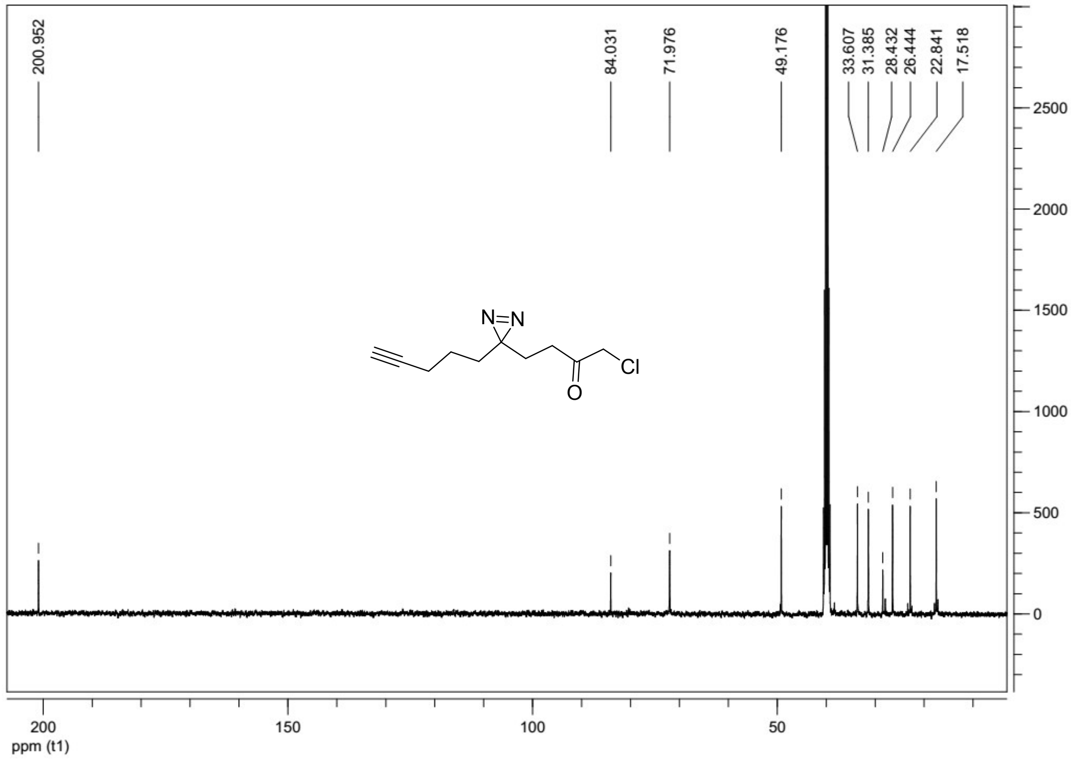

^{1}H NMR for compound **6**:

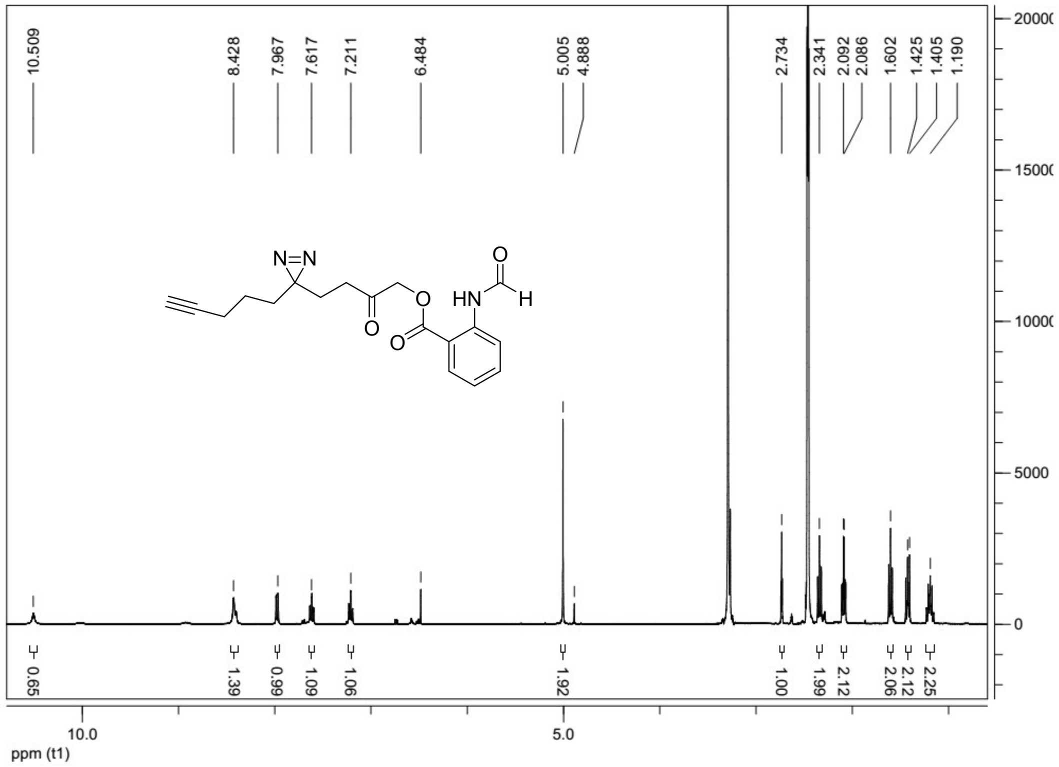

^{13}C NMR for compound **6**:

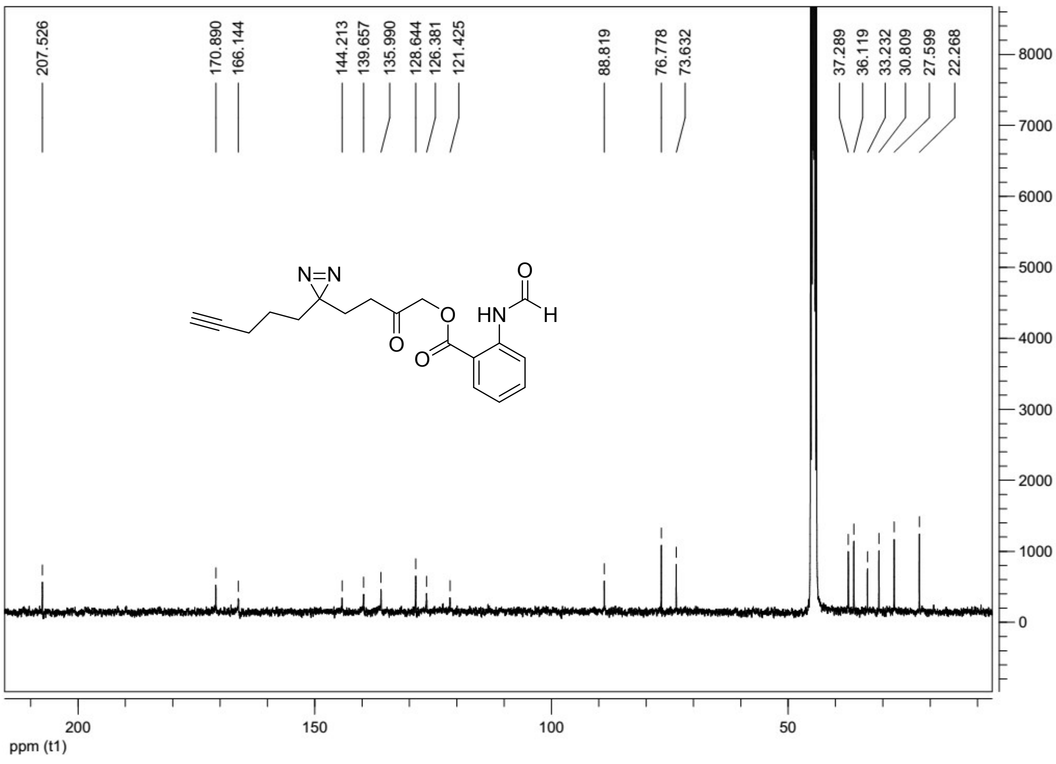

^{1}H NMR for compound **9**:

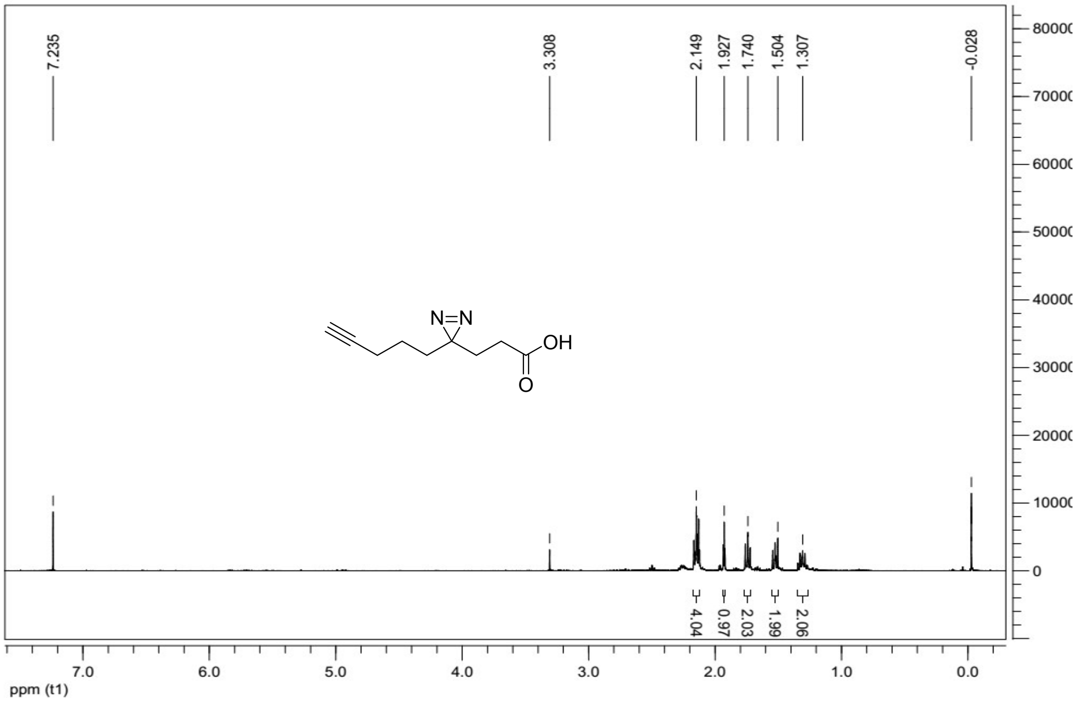

^{13}C NMR for compound **9**:

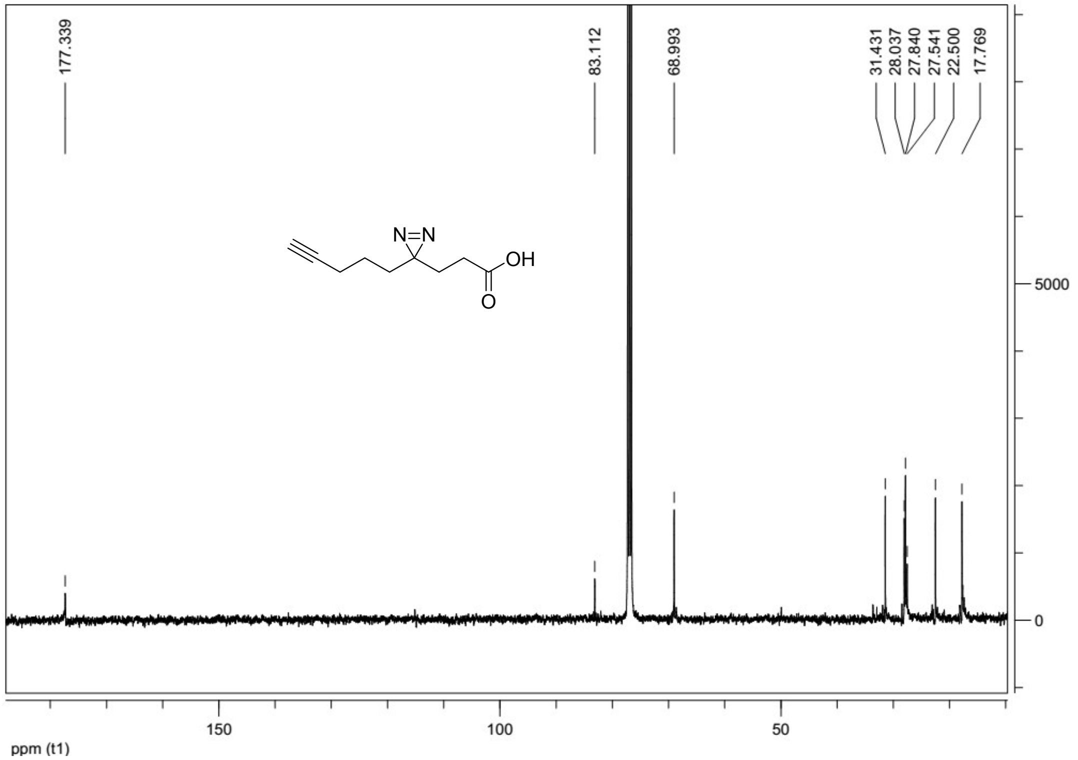

^{1}H NMR for compound **10**:

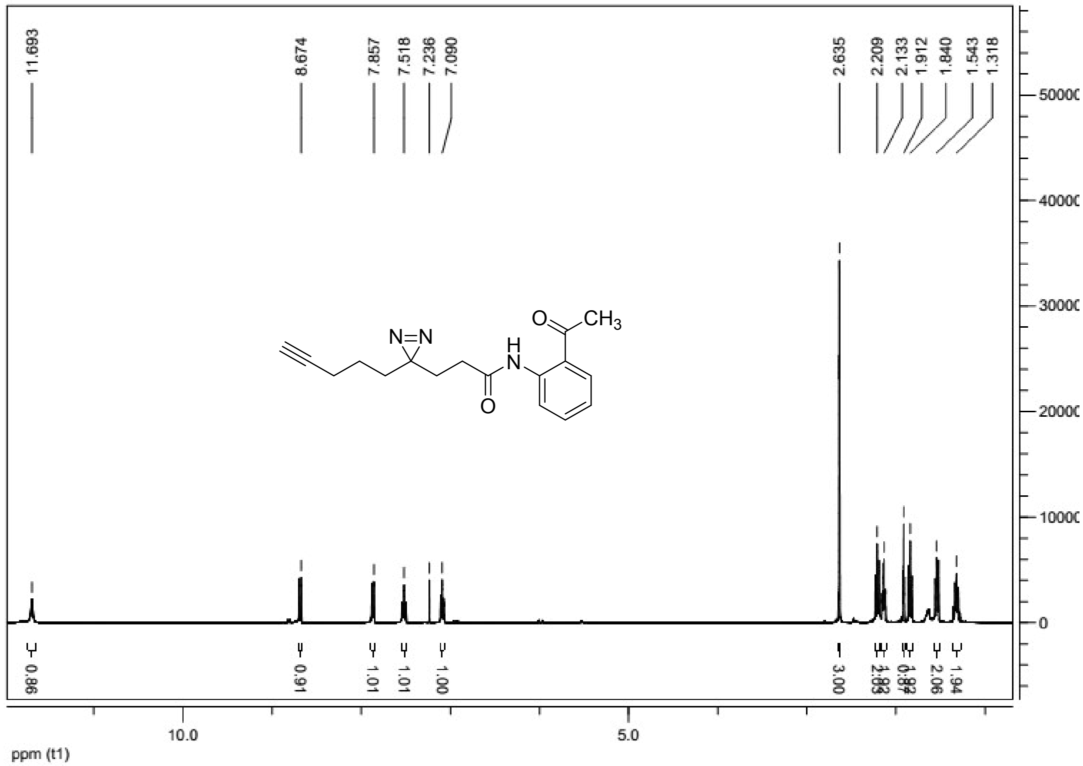

^{13}C NMR for compound **10**:

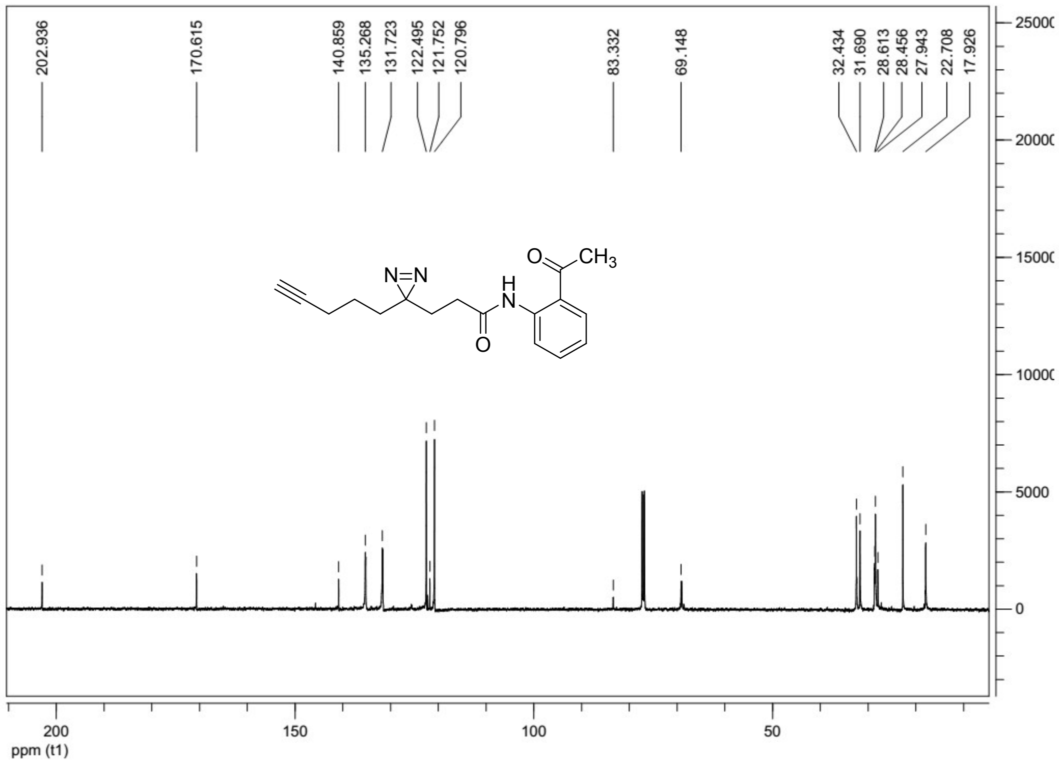

^{1}H NMR for compound **11**:

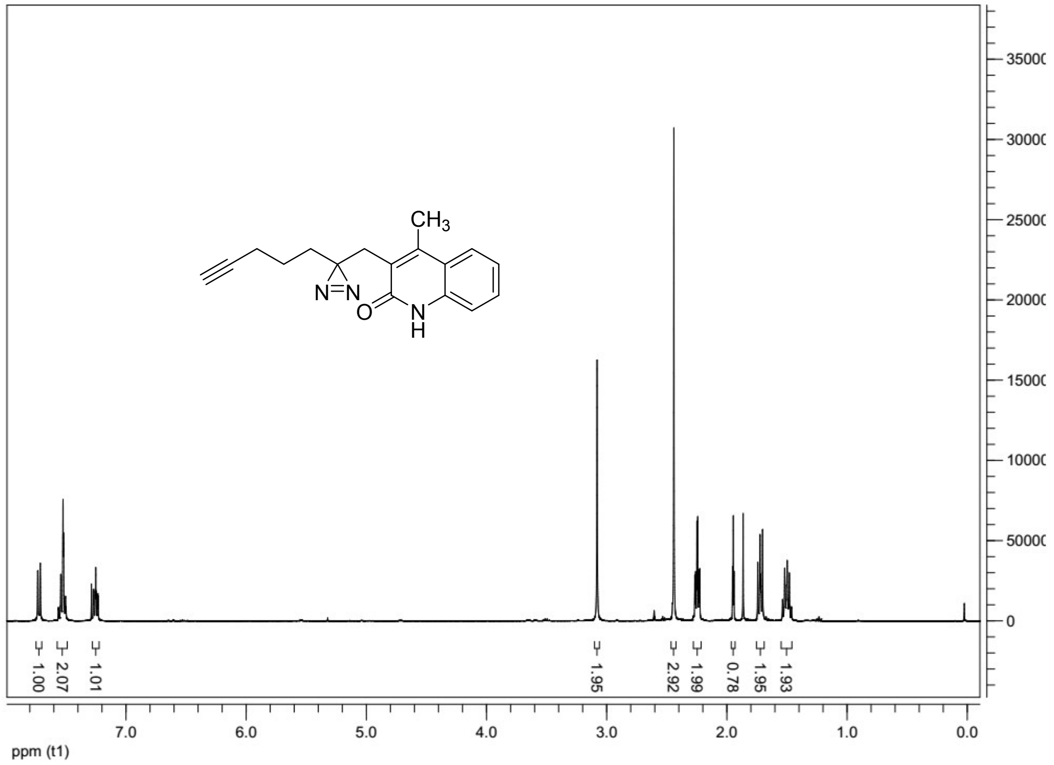

^{13}C NMR for compound **11**:

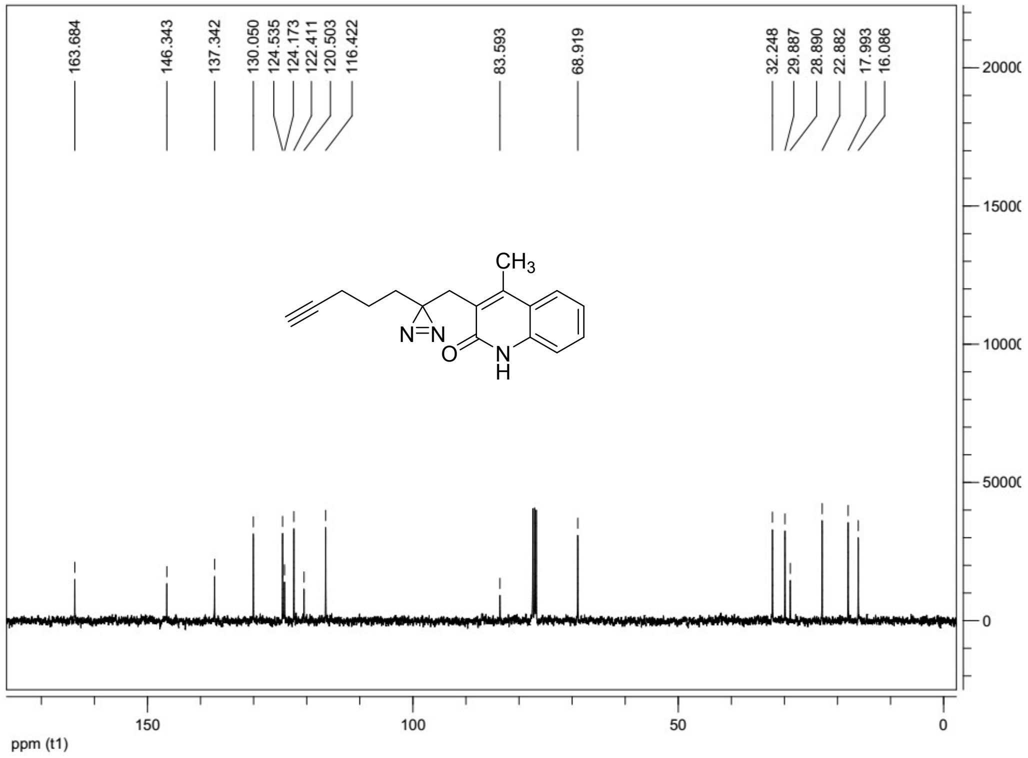

^{1}H NMR for compound **12**:

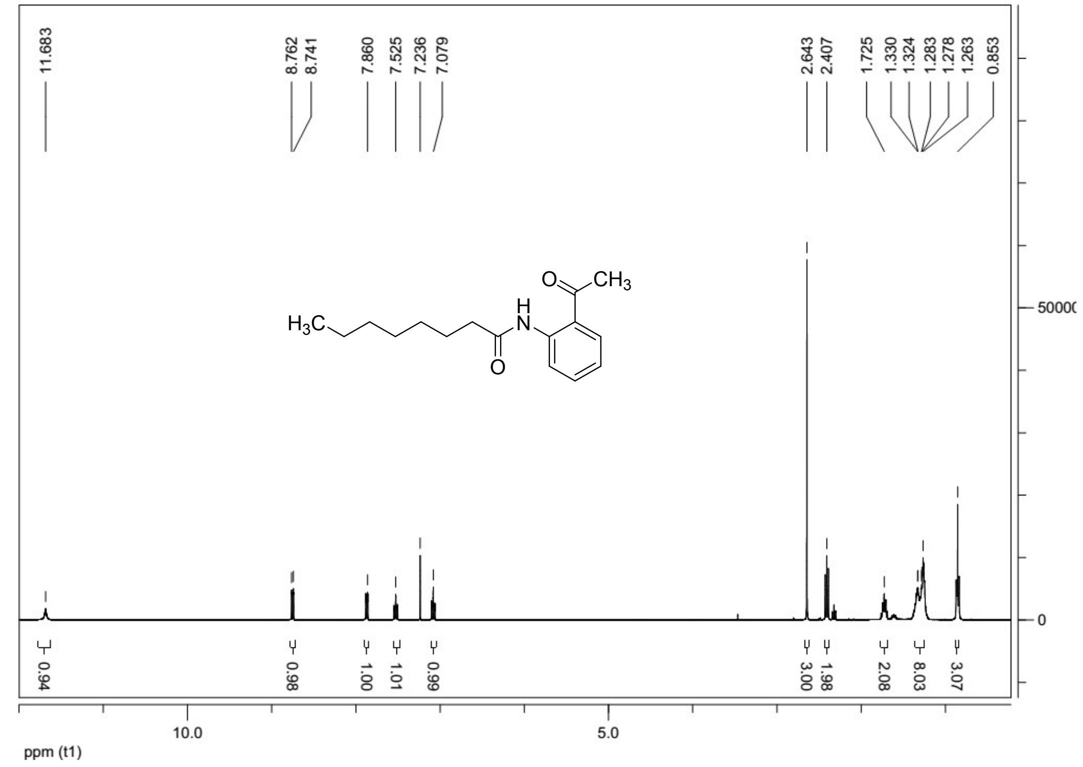

^{13}C NMR for compound **12**:

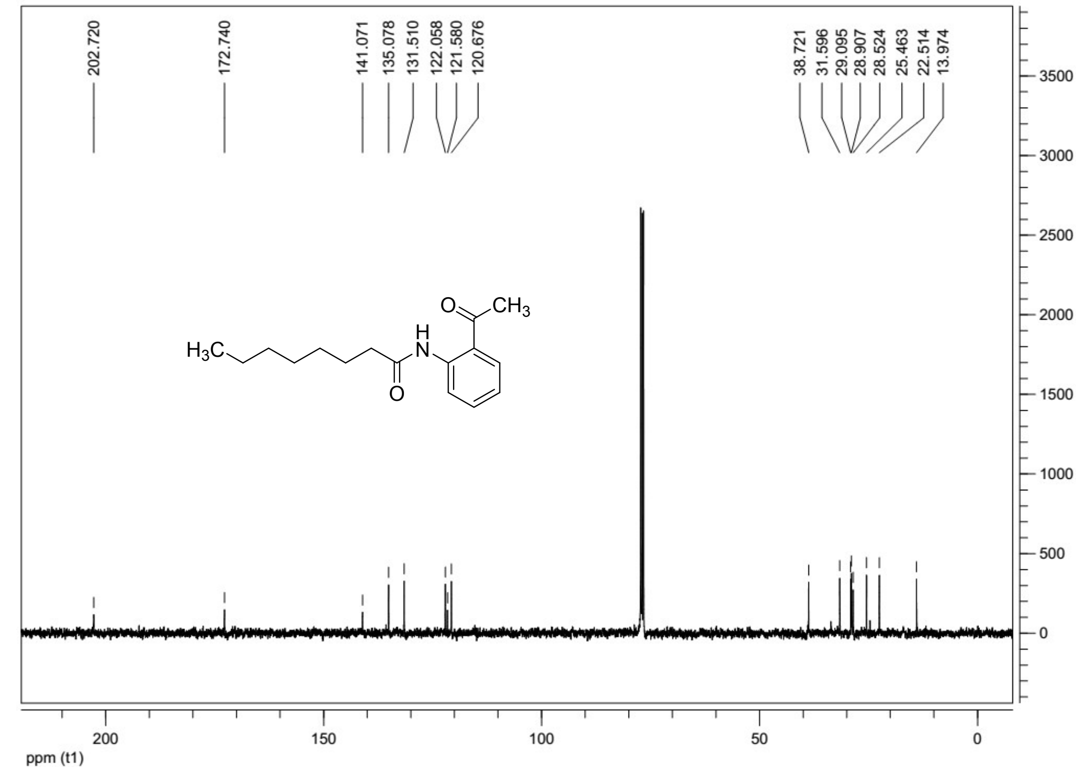

^{1}H NMR for compound **13:**

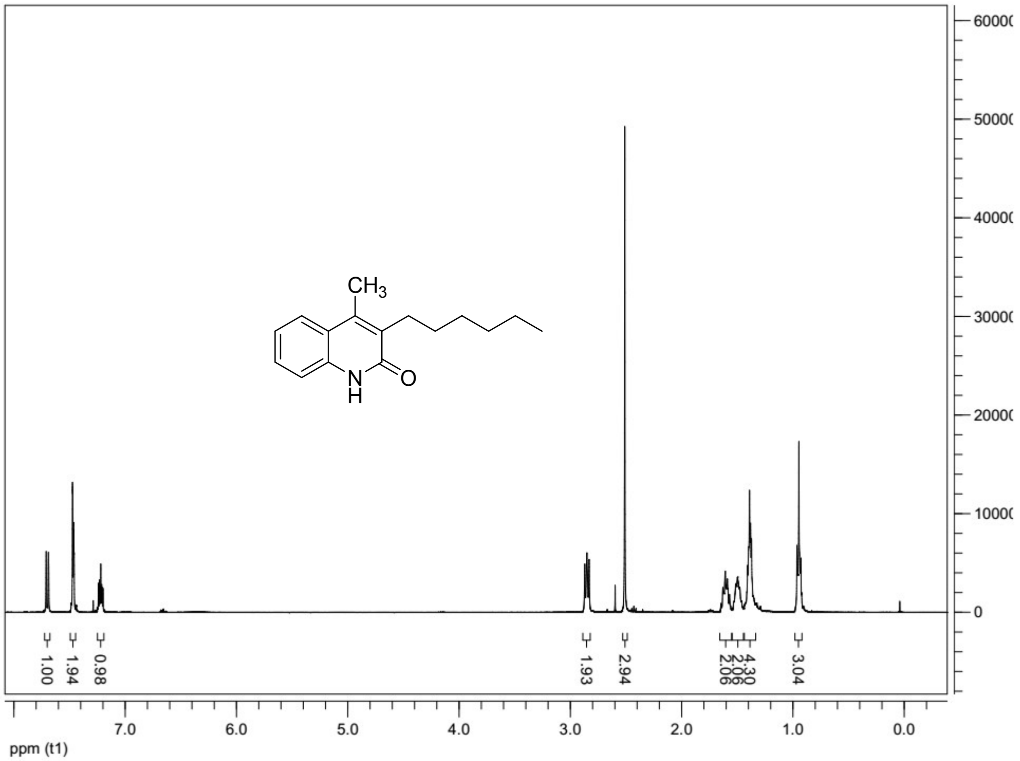

^{13}C NMR for compound **13:**

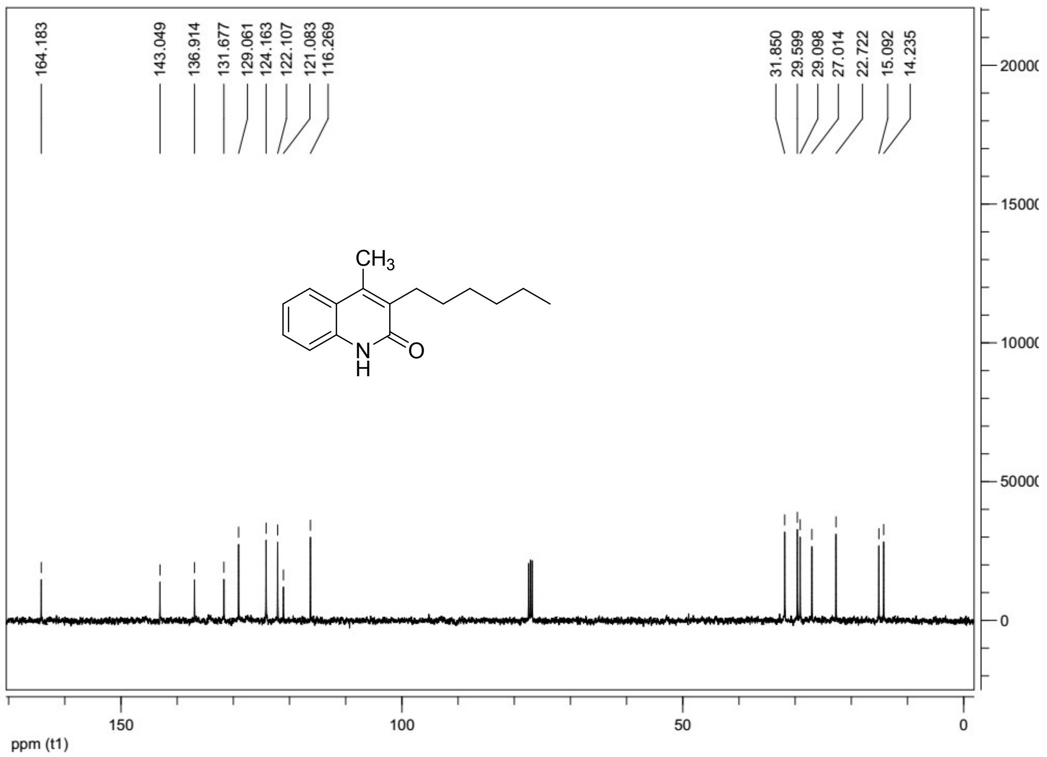

^{1}H NMR for **PQS**:

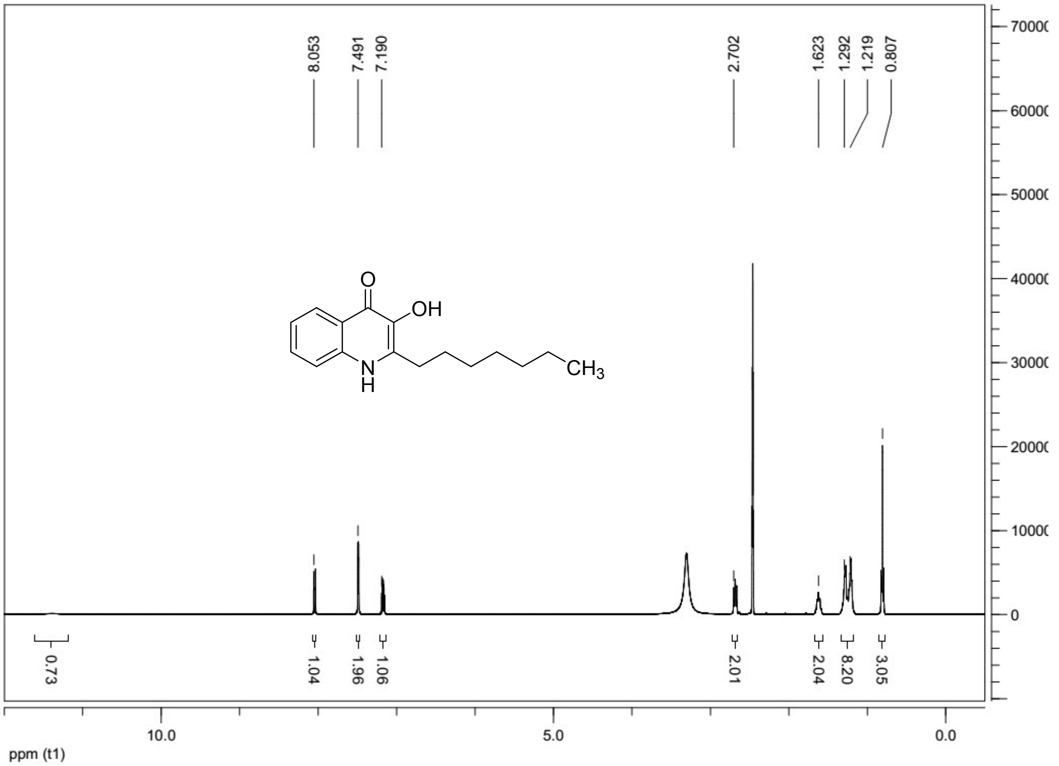

^{13}C NMR for **PQS**:

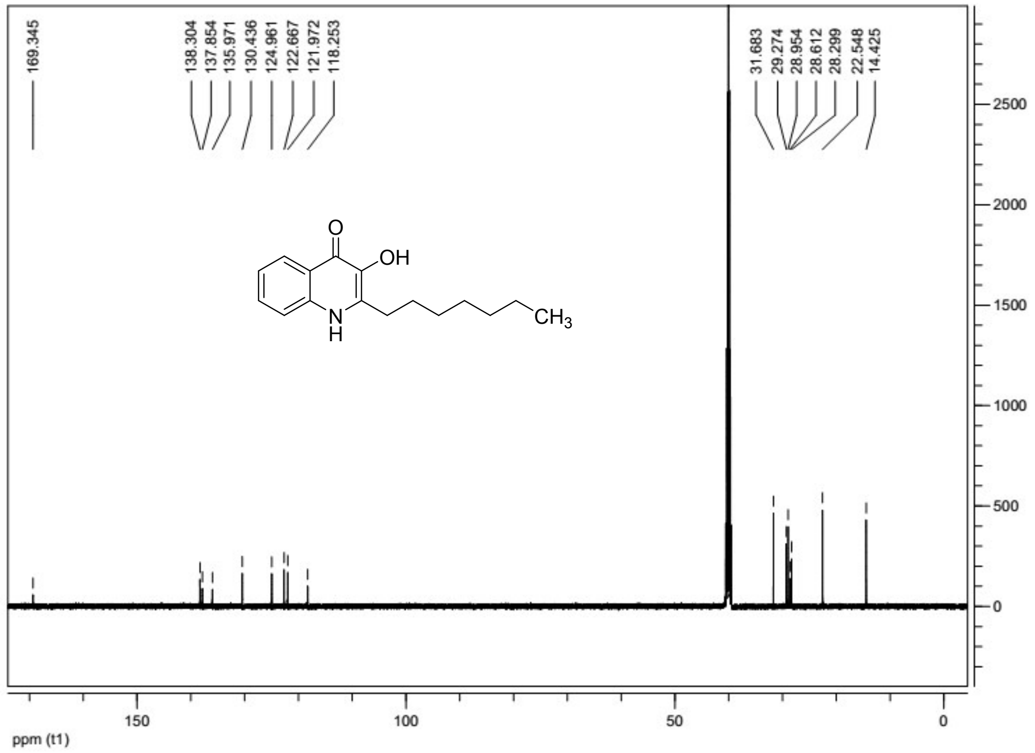

^{1}H NMR for **HHQ**:

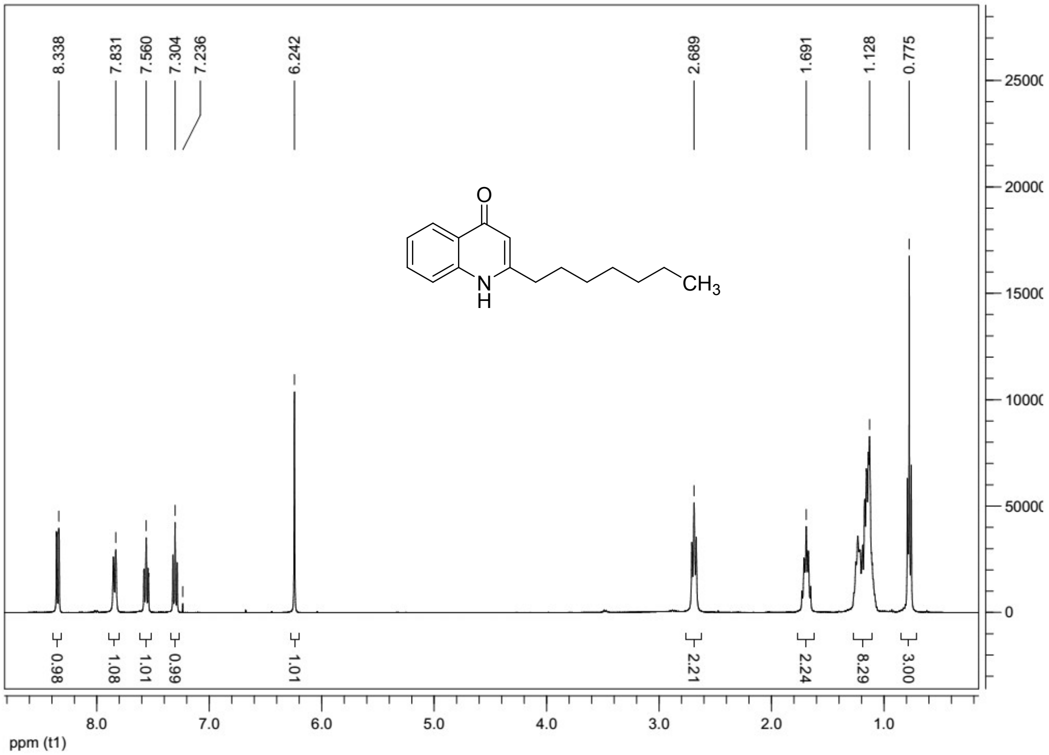

^{13}C NMR for **HHQ**:

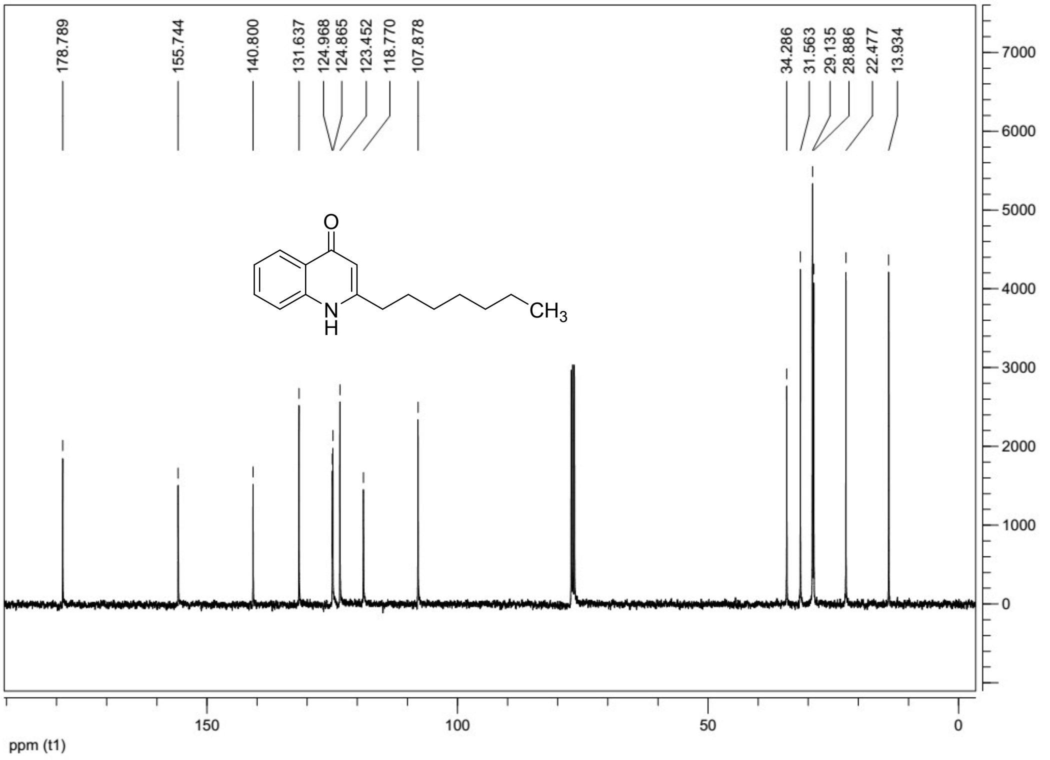

LCMS of compound **1:**

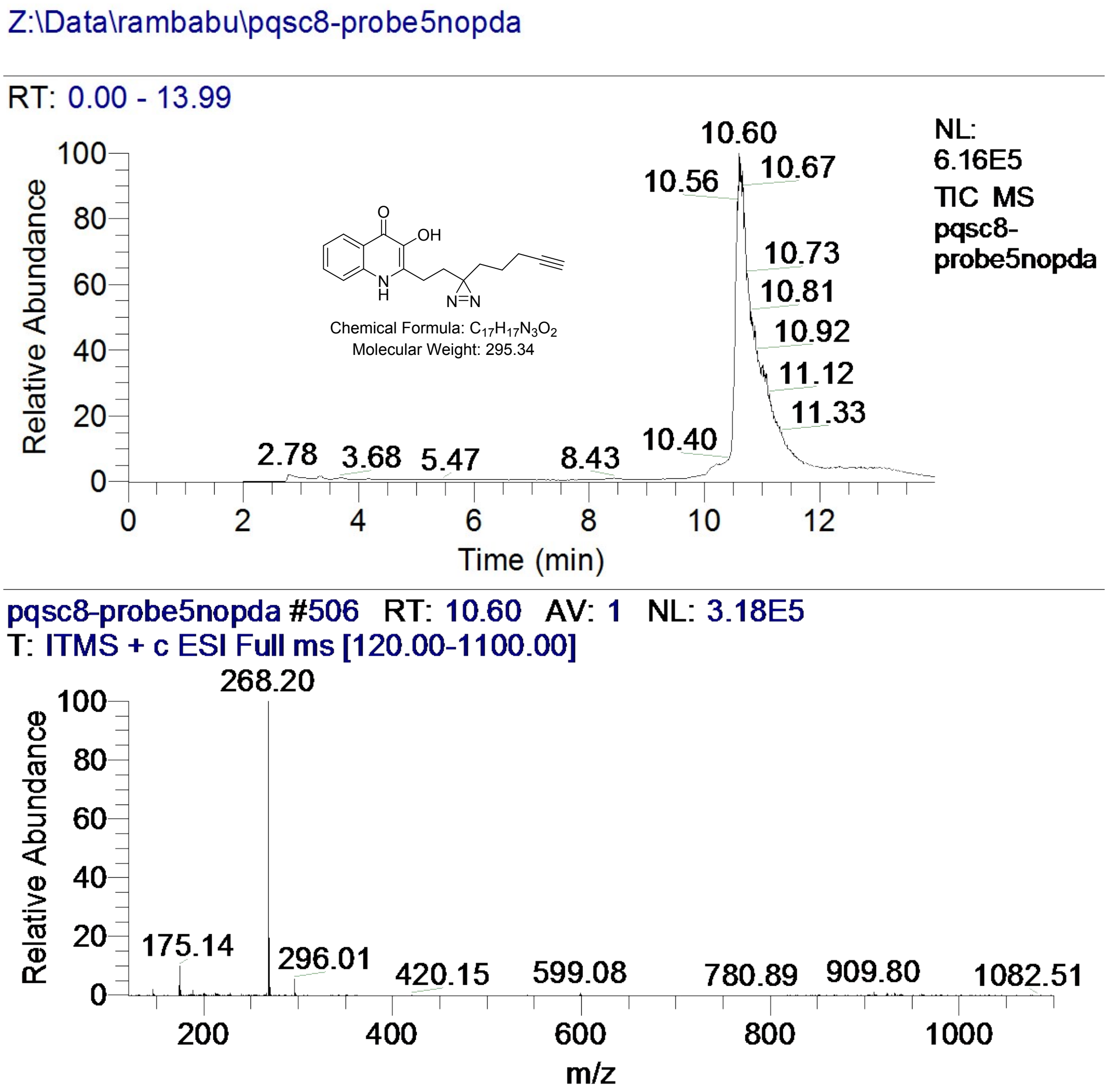

LCMS of compound **2:**

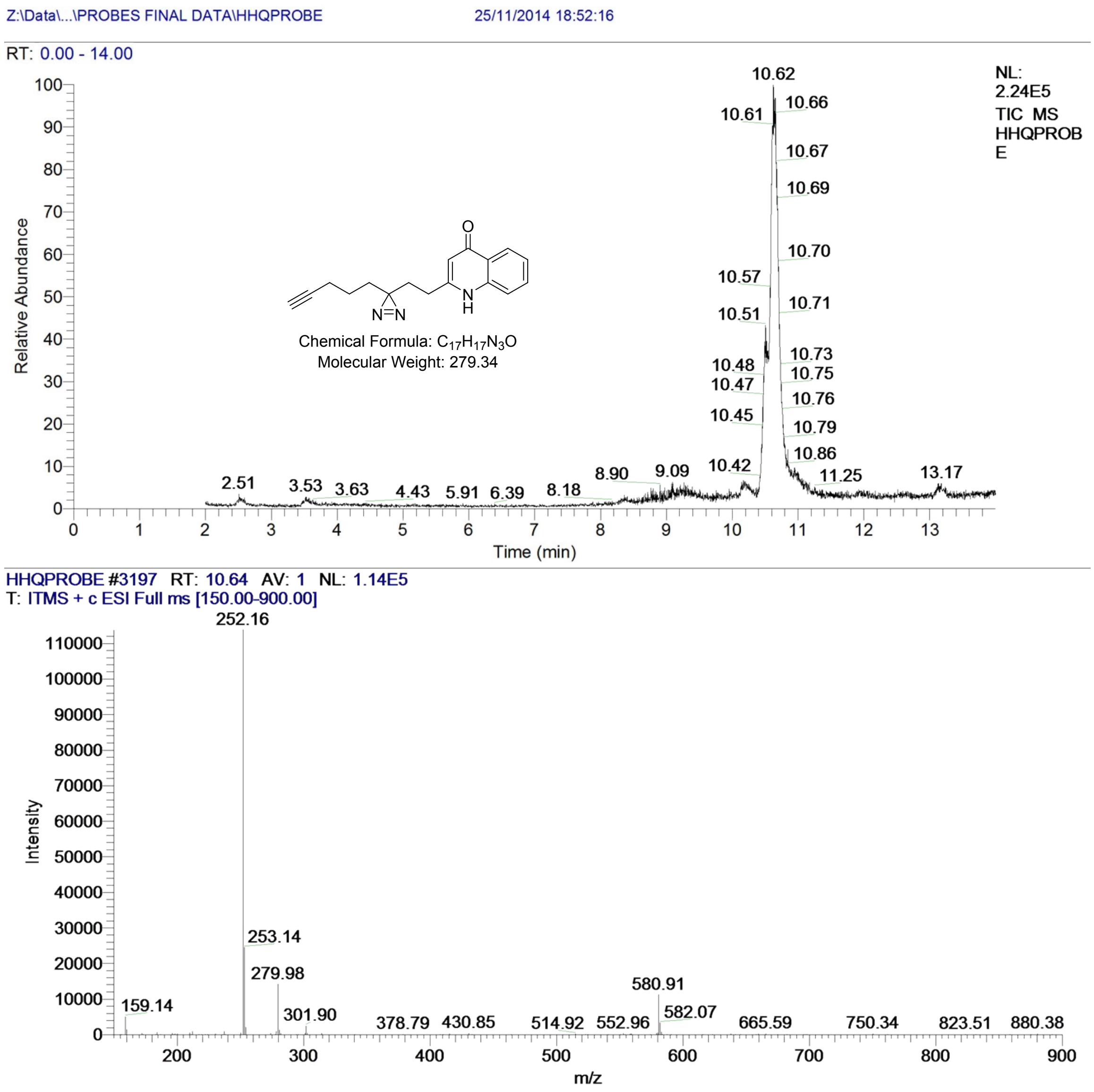

LCMS of compound **12:**

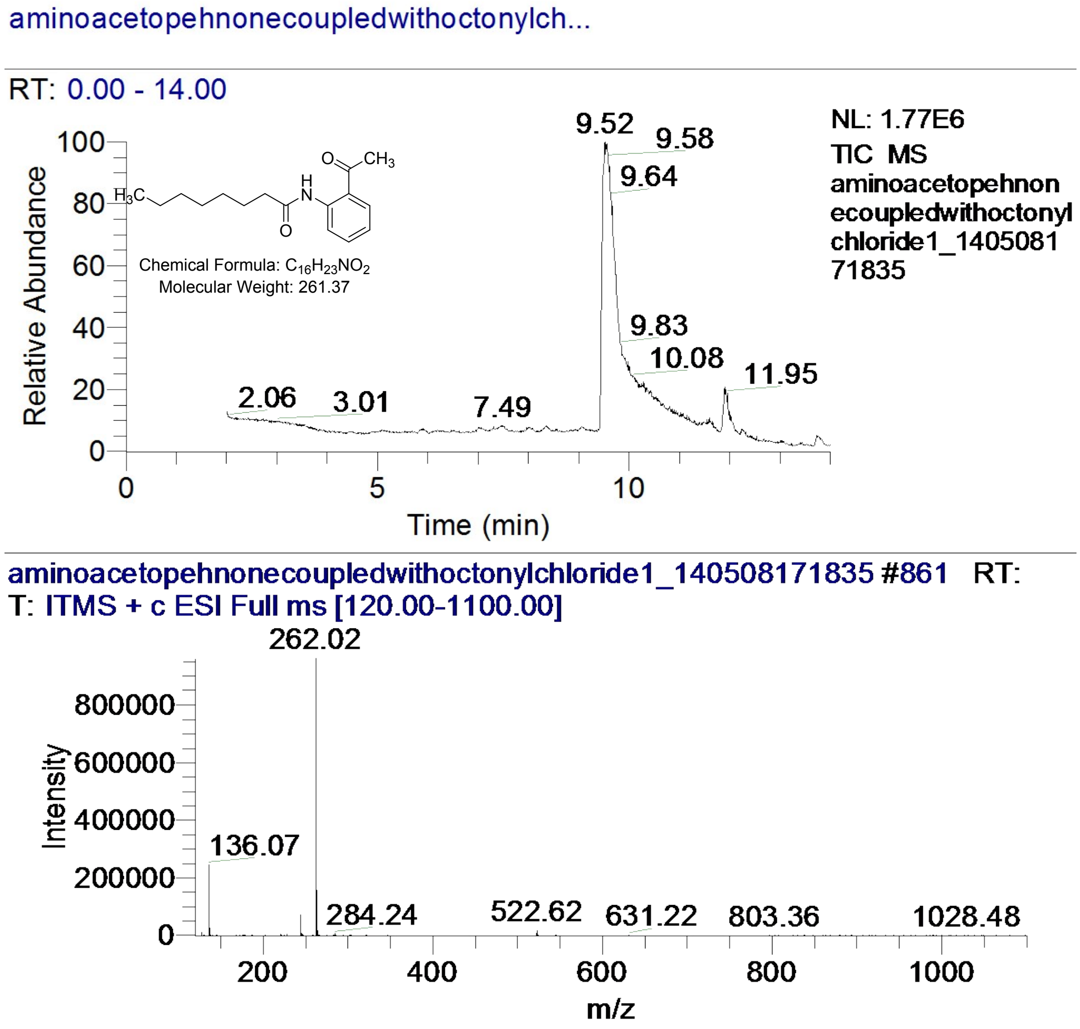

LCMS of PQS**:**

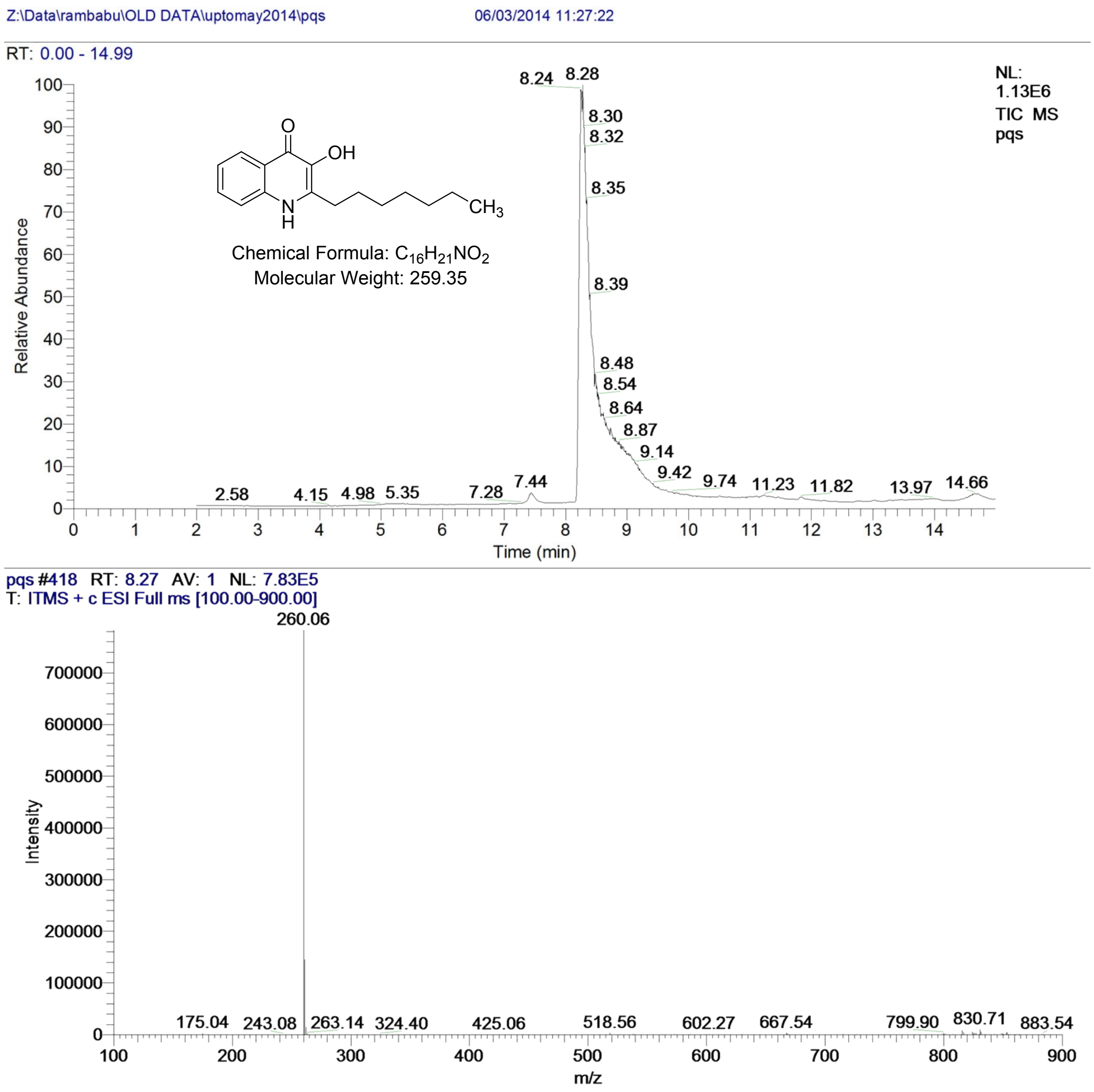

Z:\Data\rambabu\OLD DATA\uptomay2014\pqs
06/03/2014 11:27:22
RT: 0.00 - 14.99
NL: 1.13E6
TIC MS pqs
Chemical Formula: $C_{16}H_{21}NO_2$
Molecular Weight: 259.35
Relative Abundance
Time (min)
8.24 8.28 8.30 8.32 8.35 8.39 8.48 8.54 8.64 8.87 9.14 9.42 9.74 11.23 11.82 13.97 14.66
2.58 4.15 4.98 5.35 7.28 7.44
pqs #418 RT: 8.27 AV: 1 NL: 7.83E5
T: ITMS + c ESI Full ms [100.00-900.00]
260.06
175.04 243.08 263.14 324.40 425.06 518.56 602.27 667.54 799.90 830.71 883.54
Intensity
m/z

LCMS of HHQ**:**

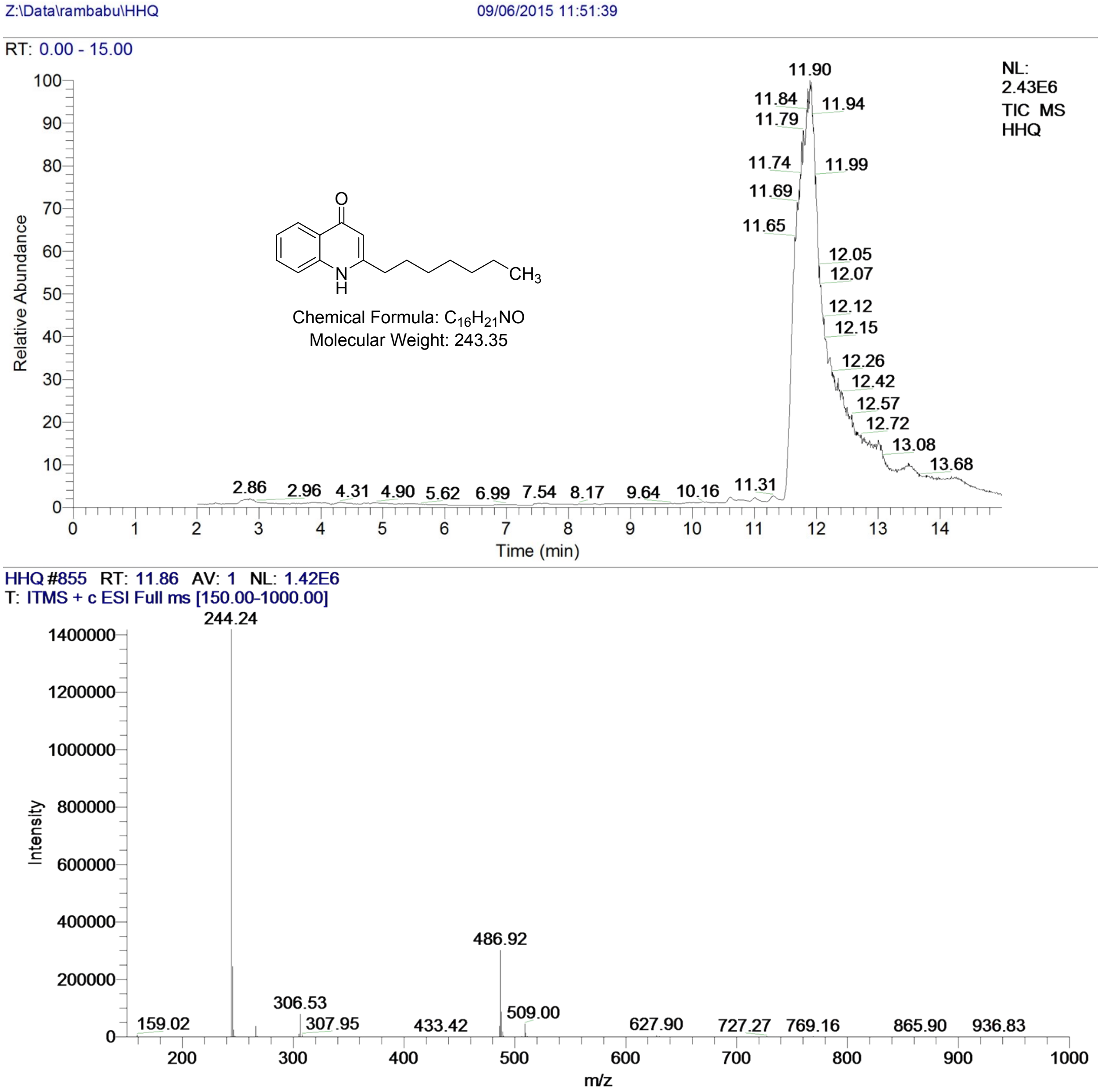
Z:\Data\rambabu\HHQ
09/06/2015 11:51:39
RT: 0.00 - 15.00
NL: 2.43E6
TIC MS HHQ
Relative Abundance
Time (min)
11.90
11.84
11.94
11.79
11.74
11.99
11.69
11.65
12.05
12.07
12.12
12.15
12.26
12.42
12.57
12.72
13.08
13.68
2.86
2.96
4.31
4.90
5.62
6.99
7.54
8.17
9.64
10.16
11.31
O
N
H
CH3
Chemical Formula: C16H21NO
Molecular Weight: 243.35
HHQ #855 RT: 11.86 AV: 1 NL: 1.42E6
T: ITMS + c ESI Full ms [150.00-1000.00]
Intensity
m/z
244.24
486.92
306.53
307.95
159.02
433.42
509.00
627.90
727.27
769.16
865.90
936.83
